# Distinct Molecular Relationships and Partial Pathway Convergence of Anserine and RRR-α-Tocopherol in Human Diabetic Kidney Disease

**DOI:** 10.3390/ijms27209279

**Published:** 2026-10-19

**Authors:** Rami M. Mosaoa

**Affiliations:** 1 Department of Biochemistry, Faculty of Science, King Abdulaziz University, Jeddah 21589, Saudi Arabia; rmosaoa@kau.edu.sa; 2 Center of Artificial Intelligence for Precision Medicines, King Abdulaziz University, Jeddah 21589, Saudi Arabia; 3 Vascular Biology Research Unit, King Fahad Medical Research Center, King Abdulaziz University, Jeddah 21589, Saudi Arabia

**Keywords:** diabetic kidney disease, anserine, RRR-α-tocopherol, transcriptomics, compound–disease integration, pathway enrichment, single-cell RNA sequencing, drug–target relationships

## Abstract

Diabetic kidney disease (DKD) involves interacting metabolic, inflammatory, vascular, and fibrotic disturbances. This study investigated the molecular relationships of anserine and RRR-α-tocopherol with human DKD using a disease-centered computational framework in which compound–target evidence was established independently before integration with kidney transcriptomic data. Stage-associated transcription was analyzed in 36 human kidney samples spanning Control, Early DKD, and Advanced DKD, with external assessment in two independent cohorts, followed by evidence-based prioritization, pathway analysis, renal single-cell localization, and sensitivity analyses. Predefined target sets comprised 100 anserine and 111 RRR-α-tocopherol targets, with only 16 shared. Integration yielded 21 prioritized compound–DKD relationships involving 20 unique genes. Despite limited target overlap, the primary pathway analysis identified shared enrichment in Class A G protein-coupled receptor, Gαi, interleukin, and receptor tyrosine kinase signaling; only receptor tyrosine kinase signaling remained shared under conservative target-confidence restriction. The 20 genes localized across diverse tubular, immune, vascular, and stromal populations. Sensitivity analyses retained 17 of 21 relationships under stricter target-confidence criteria and showed external gene–DKD replication for 18 of 21 relationships in at least one cohort. These findings define a transparent, hypothesis-generating framework for experimental testing without implying therapeutic efficacy or a confirmed mechanism of action.

## 1. Introduction

Diabetic kidney disease (DKD) is a major complication of diabetes and a leading cause of chronic kidney disease and kidney failure. Kidney disease attributable to diabetes occurs in a substantial proportion of people with diabetes and markedly increases cardiovascular and mortality risk [1]. Although contemporary management incorporating renin–angiotensin system blockade, sodium–glucose cotransporter 2 inhibitors, glucagon-like peptide-1 receptor agonists, and nonsteroidal mineralocorticoid receptor antagonists has substantially expanded kidney-protective treatment options, considerable residual risk remains. This persistent burden reflects, in part, the biological complexity of DKD, in which metabolic and hemodynamic disturbances interact with oxidative, inflammatory, vascular, and fibrotic processes rather than operating through a single pathogenic pathway [2].

Human transcriptomic studies have increasingly demonstrated that this complexity is accompanied by substantial molecular remodeling across disease stages and renal cellular compartments. Bulk kidney transcriptomic analyses have identified stage- and compartment-associated alterations involving metabolic, immune, inflammatory, and extracellular-matrix programs, while single-cell and single-nucleus approaches have revealed heterogeneous responses across tubular, endothelial, stromal, and immune populations [3,4,5]. These observations provide an opportunity to evaluate candidate compounds in relation to the molecular architecture of human DKD rather than relying exclusively on predefined disease mechanisms or individual experimental models. Integrating independently established compound–target relationships with human disease transcriptomics may therefore help distinguish molecular associations that intersect reproducible DKD biology from those supported only by compound-centered evidence.

Anserine is a naturally occurring histidine-containing dipeptide related to carnosine and has been investigated for antioxidant, carbonyl-scavenging, and antiglycation properties. Histidine-containing dipeptides can interact with reactive carbonyl species generated during oxidative and glycative stress, biological processes that are highly relevant to diabetes and its complications [6,7]. Experimental evidence specifically addressing anserine in diabetic kidney injury remains comparatively limited. In human renal tubular cells exposed to high glucose, anserine enhanced cellular stress-response pathways and reduced protein carbonylation, while short-term administration in diabetic mice reduced proteinuria and vascular permeability and improved glycemic measures [8]. These findings support potential biological relevance in diabetic renal injury but leave the molecular relationships connecting anserine to human DKD incompletely characterized.

RRR-α-tocopherol is the naturally occurring stereoisomer of α-tocopherol. α-Tocopherol is preferentially retained in the body through selective metabolic handling involving the α-tocopherol transfer protein [9]. Although best established as a lipid-soluble antioxidant that protects membranes from lipid peroxidation, α-tocopherol has also been reported to exert regulatory effects on cellular signaling and enzyme activity [10]. Vitamin E has also been investigated clinically in diabetic nephropathy. In a randomized, placebo-controlled trial, high-dose vitamin E supplementation reduced urinary protein measures and several inflammatory and oxidative-stress biomarkers, although the specific α-tocopherol stereoisomer administered was not reported [11]. Of particular relevance, previous experimental work evaluated anserine and α-tocopherol individually and in combination in streptozotocin-induced diabetic rats and reported improvements in glycemic indices and in markers of oxidative stress, inflammation, and advanced glycation end products [12]. Together, these observations provide a biological rationale for considering anserine and α-tocopherol in the context of diabetic kidney injury, while leaving unresolved whether their molecular relationships with diabetic kidney injury involve shared targets, distinct biological processes, or convergence at higher levels of disease organization.

The present study therefore investigated the molecular relationships of anserine and the stereochemically defined RRR-α-tocopherol within human DKD using an integrative, disease-centered computational framework. Compound–target evidence was established independently of the disease analyses and subsequently integrated with stage-resolved human kidney transcriptomics to identify prioritized compound–DKD relationships supported by predefined evidence configurations. Independent human DKD cohorts were used to assess the reproducibility of gene–disease associations, while pathway analysis and renal single-cell localization were applied subsequently to provide biological context rather than to generate additional gene-level priorities. The study was designed to determine whether the largely distinct target profiles of the two compounds intersect reproducible components of the human DKD molecular landscape and whether they show convergence at higher levels of biological organization. Rather than inferring therapeutic efficacy or mechanism from computational association alone, the objective was to define a transparent set of experimentally testable compound–DKD relationships for subsequent mechanistic investigation.

## 2. Results

**Figure 1:**
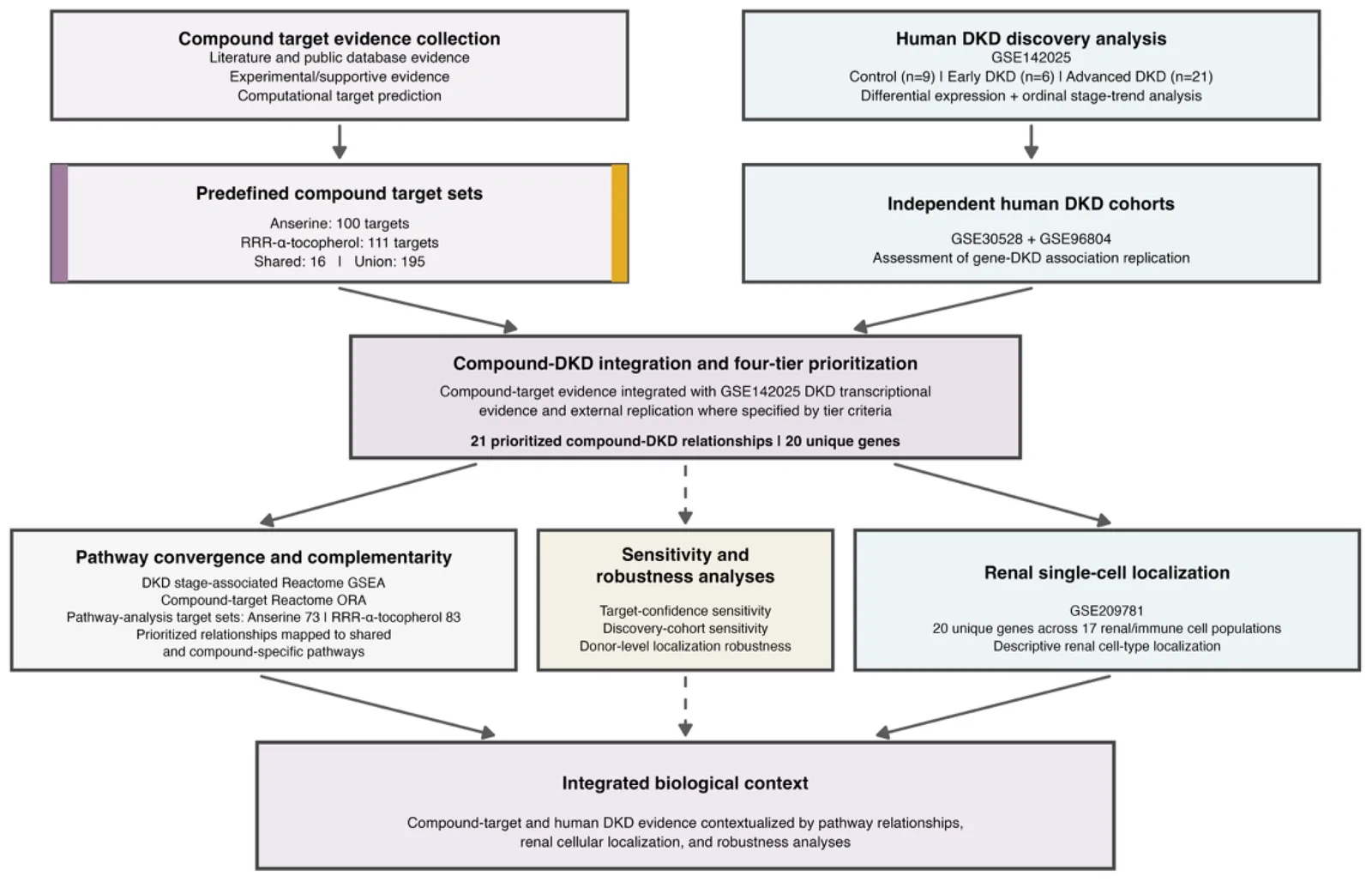
Study design integrating predefined compound–target evidence with human diabetic kidney disease (DKD) analyses. Compound–target evidence for anserine and RRR-α-tocopherol was assembled independently from the literature, curated databases, experimental or supportive evidence, and computational target-prediction resources to establish predefined target sets of 100 and 111 targets, respectively. In parallel, cross-sectional stage-associated transcriptional changes were characterized in GSE142025 across Control (*n* = 9), Early DKD (*n* = 6), and Advanced DKD (*n* = 21), with gene–DKD association replication assessed in the independent human DKD cohorts GSE30528 and GSE96804. Compound–target evidence was then integrated with the human DKD transcriptional evidence using the predefined four-tier prioritization framework, yielding 21 prioritized compound–DKD relationships involving 20 unique genes. These prioritized relationships constituted the terminal gene-level result and were established before downstream pathway and single-cell analyses. Pathway context was evaluated separately using DKD stage-associated Reactome gene-set enrichment analysis and compound–target Reactome over-representation analysis based on pathway-analysis target sets of 73 anserine and 83 RRR-α-tocopherol targets; the prioritized relationships were subsequently mapped to shared and compound-specific pathway results for interpretation. The 20 unique prioritized genes were additionally localized descriptively across 17 renal and immune cell populations in GSE209781. Supporting sensitivity and robustness analyses assessed target-confidence restriction, discovery-cohort robustness, and donor-level stability of renal localization. Together, these complementary analyses provided integrated biological context for the prioritized relationships without introducing a further gene-selection step. Solid arrows denote the principal analytical workflow, whereas dashed arrows denote supporting sensitivity and robustness analyses. Compound–target evidence and human DKD evidence were treated as distinct evidence dimensions throughout the workflow.

The study integrated predefined compound–target evidence for anserine and RRR-α-tocopherol with stage-resolved human DKD transcriptomic analysis, external assessment in independent human DKD cohorts, pathway-level analyses, and renal single-cell localization (Figure 1). Compound–target evidence and human DKD evidence were maintained as distinct evidence dimensions throughout the analysis. Integration of the predefined compound–target sets with the human DKD transcriptional evidence yielded 21 prioritized compound–DKD relationships representing 20 unique genes, which constituted the terminal gene-level result. Pathway and single-cell analyses subsequently provided biological context for these relationships, while complementary sensitivity and robustness analyses evaluated target-confidence, discovery-cohort, external disease replication, and renal-localization stability without introducing a further gene-selection step.

### 2.1. Predefined Compound–Target Sets Show Limited Overlap

The predefined compound–target sets, assembled from the literature, public databases, and computational target-prediction evidence, comprised 100 targets for anserine and 111 targets for RRR-α-tocopherol. Comparison of the two target universes identified 84 anserine-specific targets, 95 RRR-α-tocopherol-specific targets, and 16 shared targets, yielding 195 unique targets overall and a Jaccard similarity of 8.2% (Supplementary Data S1). The limited overlap therefore indicated largely distinct compound–target profiles for the two compounds, which were subsequently integrated separately with the human DKD transcriptomic evidence.

### 2.2. Stage-Associated Transcriptomic Landscape of Human DKD

**Figure 2:**
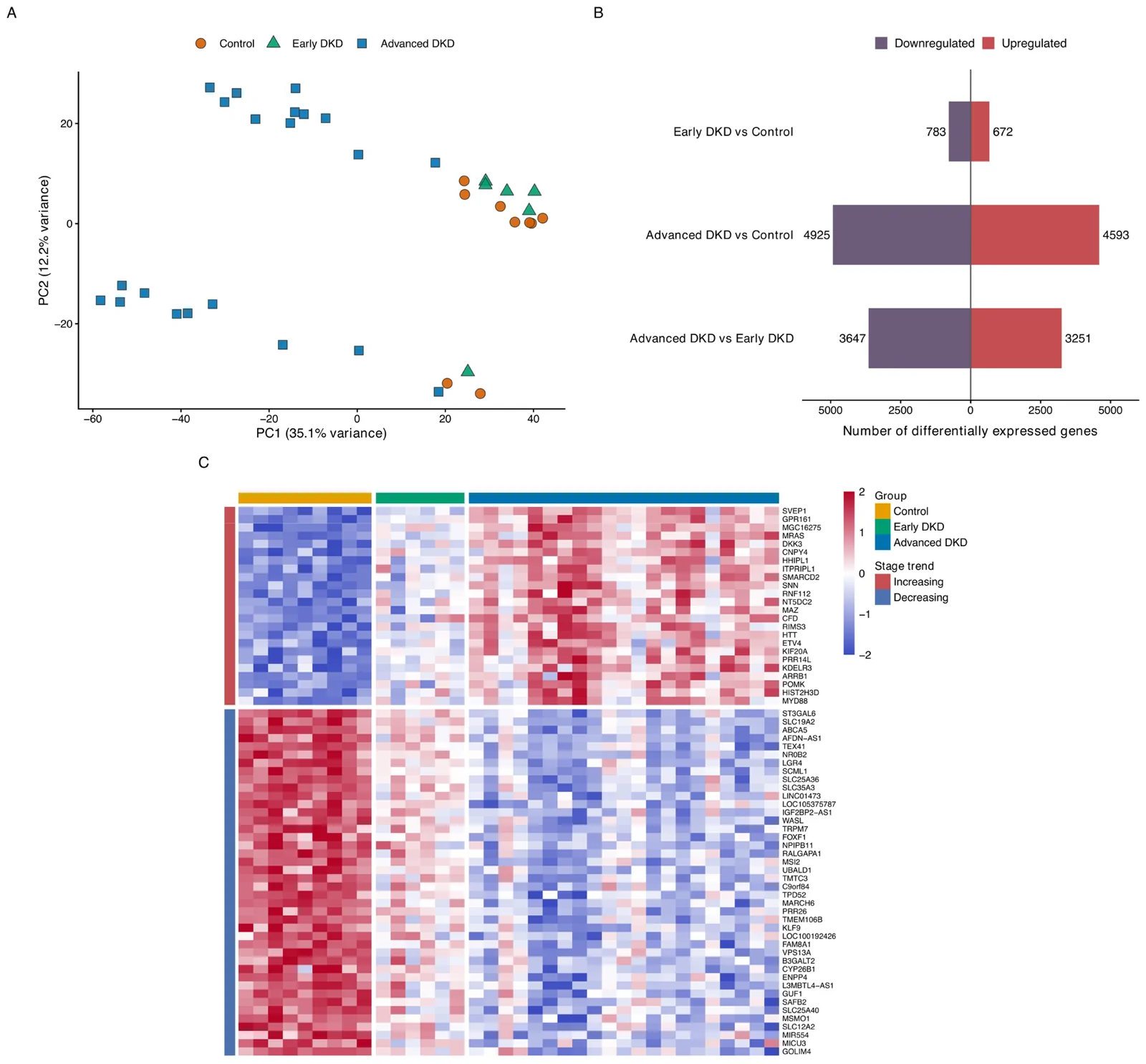
Stage-resolved transcriptional landscape of human diabetic kidney disease (DKD) in GSE142025. (**A**) Principal component analysis (PCA) of the 1000 most variable genes across 36 human kidney samples comprising Control (*n* = 9), Early DKD (*n* = 6), and Advanced DKD (*n* = 21). The first two principal components (PC1 and PC2) explain 35.1% and 12.2% of the variance, respectively. (**B**) Numbers of differentially expressed genes for Early DKD versus Control, Advanced DKD versus Control, and Advanced DKD versus Early DKD. Differential expression was defined using a Benjamini–Hochberg false discovery rate (FDR) < 0.05 without an absolute log2 fold-change threshold. Purple and red bars represent downregulated and upregulated genes, respectively. (**C**) Row-wise standardized expression heatmap of the stringent 66-gene stage-associated signature across Control, Early DKD, and Advanced DKD samples. The signature comprises 24 genes with increasing and 42 genes with decreasing expression across DKD stages. Red and blue indicate higher and lower expression, respectively, relative to each gene’s own standardized expression distribution. Genes are grouped according to predefined increasing or decreasing stage trends; no unsupervised clustering was applied. Because GSE142025 is cross-sectional, expression patterns are interpreted as stage-associated differences rather than longitudinal changes within individuals.

Principal component analysis of the 1000 most variable genes across the 36 kidney tissue samples in GSE142025, comprising Control, Early DKD, and Advanced DKD groups, showed stage-associated differences in global transcriptional profiles (Figure 2A). The first two principal components explained 35.1% and 12.2% of the total variance, respectively. Advanced DKD samples showed greater displacement and heterogeneity, whereas Early DKD and Control samples exhibited partial overlap. Differential expression analysis further revealed substantial transcriptional differences across pairwise group comparisons (Figure 2B). At a Benjamini–Hochberg false discovery rate (FDR) < 0.05, 1455 genes were differentially expressed in Early DKD versus Control, including 672 upregulated and 783 downregulated genes. Advanced DKD versus Control yielded 9518 differentially expressed genes, comprising 4593 upregulated and 4925 downregulated genes, while Advanced DKD versus Early DKD identified 6898 differentially expressed genes, including 3251 upregulated and 3647 downregulated genes.

Ordinal-stage analysis identified 9613 genes with significant stage-associated expression differences, of which 5841 showed monotonic patterns across Control, Early DKD, and Advanced DKD, including 2759 genes with increasing and 3082 with decreasing expression. The remaining 3772 significant stage-associated genes exhibited non-monotonic patterns. Applying stringent criteria requiring a significant ordinal-stage trend, monotonic expression across the three groups, significant differences in both adjacent-stage comparisons, and directionally consistent changes yielded a 66-gene stage-associated signature comprising 24 genes with increasing and 42 with decreasing expression (Figure 2C). The heatmap of this stringent signature demonstrated coordinated stage-associated expression patterns across the three groups.

### 2.3. External Validation of the Stringent Stage-Associated DKD Signature

Genome-wide effect-size concordance with the GSE142025 Advanced DKD-versus-Control comparison was modestly positive in both external cohorts, with Pearson correlations of r = 0.304 for GSE30528 and r = 0.373 for GSE96804. Among genes reaching FDR < 0.05 in both the discovery and corresponding validation analyses, 868 of 1193 genes (72.76%) in GSE30528 and 3249 of 4260 genes (76.27%) in GSE96804 showed concordant directions of effect. The 66-gene stage-associated signature was evaluated in two independent human DKD cohorts, GSE30528 and GSE96804. In GSE30528, 44 of the 66 genes were measurable, 15 were significantly differentially expressed, and 12 met the predefined external-replication criterion of FDR < 0.05 with an effect direction concordant with GSE142025 Advanced DKD-versus-Control contrast (Supplementary Figure S1A; Supplementary Table S1). In GSE96804, 58 genes were measurable, 29 were significantly differentially expressed, and 23 met the same replication criterion (Supplementary Figure S1B; Supplementary Table S1). Five genes, *SVEP1*, *KIF20A*, *TPD52*, *KLF9*, and *GOLIM4*, met the replication criterion in both external cohorts.

### 2.4. Integration of Compound–Target Profiles with Human DKD Transcriptional Evidence

**Figure 3:**
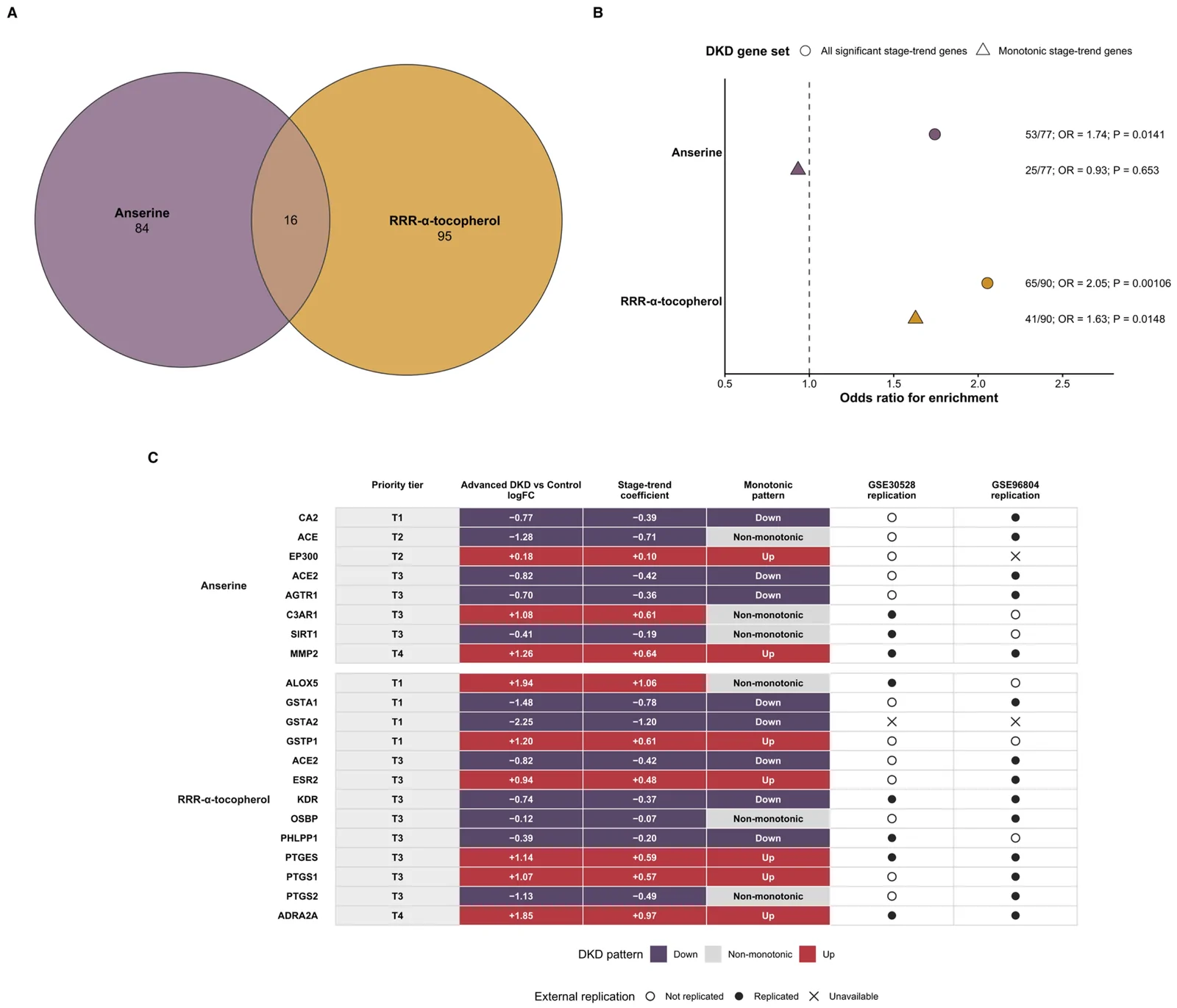
Compound–target architecture and integration with human diabetic kidney disease (DKD) transcriptional evidence. (**A**) Proportional overlap of the predefined compound–target sets for anserine and RRR-α-tocopherol. The predefined target sets comprised 100 targets for anserine and 111 targets for RRR-α-tocopherol, including 16 shared targets and a union of 195 unique targets (Jaccard similarity, 8.2%). (**B**) Enrichment of measurable compound targets among genes showing significant ordinal DKD stage associations and among the subset showing monotonic stage-associated expression patterns in GSE142025. Circles denote all significant stage-associated genes and triangles denote monotonic stage-associated genes; the dashed vertical line indicates an odds ratio (OR) of 1. Enrichment was evaluated using one-sided Fisher exact tests. No formal statistical comparison between the two compounds was performed. (**C**) Integrated evidence matrix for the 21 prioritized compound–DKD relationships, representing 20 unique genes because *ACE2* was prioritized for both compounds. Columns indicate compound–target priority tier, Advanced DKD-versus-Control log2 fold change, ordinal-stage-association coefficient, monotonic expression pattern, and external disease-cohort replication in GSE30528 and GSE96804. Red, purple, and gray denote increasing, decreasing, and non-monotonic stage-associated expression patterns, respectively. For external replication, filled circles indicate genes meeting the predefined criterion of false discovery rate (FDR) < 0.05 in the external DKD-versus-Control comparison with effect direction concordant with GSE142025 Advanced DKD versus Control; open circles indicate genes for which external evidence was available but the criterion was not met; crosses indicate unavailable external evidence. External replication refers to the gene–DKD association and not to validation of the corresponding compound–target relationship. Priority tiers represent predefined configurations of compound–target and human DKD evidence rather than a numerical composite score or a simple strongest-to-weakest ranking, and prioritization preceded downstream pathway and single-cell analyses.

Before assessing enrichment of compound targets within the DKD stage-associated transcriptional landscape, the relationship between the predefined target sets was visualized. Consistent with the target-set comparison described above, anserine and RRR-α-tocopherol showed limited overlap, with 84 anserine-specific targets, 95 RRR-α-tocopherol-specific targets, and 16 shared targets (Figure 3A). Of the full predefined target sets, 77 of 100 anserine targets and 90 of 111 RRR-α-tocopherol targets were measurable in GSE142025 and were therefore carried forward into the transcriptomic enrichment analysis.

Among targets measurable in GSE142025, anserine targets were significantly enriched among genes showing a significant ordinal-stage trend, with 53 of 77 targets meeting this criterion (odds ratio [OR] = 1.74, *p* = 0.014). RRR-α-tocopherol targets were also significantly enriched among stage-trend genes, with 65 of 90 targets meeting the criterion (OR = 2.05, *p* = 0.001; Figure 3B). When the analysis was restricted to genes showing monotonic expression patterns across Control, Early DKD, and Advanced DKD, 25 of 77 anserine targets were represented, with no significant enrichment (OR = 0.93, *p* = 0.653), whereas 41 of 90 RRR-α-tocopherol targets were represented and showed significant enrichment (OR = 1.63, *p* = 0.015).

Integration of the predefined compound–target evidence with the human DKD evidence yielded 21 prioritized compound–DKD relationships, comprising eight relationships for anserine and 13 for RRR-α-tocopherol (Figure 3C; Supplementary Table S2). The relationships were classified using the investigator-defined four-tier evidence framework, which preserved distinct configurations of compound–target and human DKD evidence rather than combining them into a numerical composite score. Tier 1 comprised human-experimental DKD candidates, Tier 2 supportive translational candidates, Tier 3 balanced computational priorities, and Tier 4 disease-led computational priorities. Across the 21 relationships, five were assigned to Tier 1, two to Tier 2, 12 to Tier 3, and two to Tier 4. The Tier 1 relationships comprised *CA2* for anserine and *ALOX5, GSTA1, GSTA2*, and *GSTP1* for RRR-α-tocopherol. Figure 3C additionally summarizes the Advanced-DKD-versus-Control expression change, ordinal-stage association, monotonicity, and external disease-replication status of each relationship. The tier configurations were not interpreted as a simple strongest-to-weakest ranking. The 21 relationships, representing 20 unique genes because *ACE2* was prioritized independently for both compounds, constituted the terminal gene-level result and were established before the downstream pathway and single-cell analyses.

### 2.5. Pathway-Level Convergence and Compound-Specific DKD-Associated Pathway Relationships

Disease-first Hallmark gene-set enrichment analysis (GSEA) identified 31 of 50 Hallmark gene sets as significantly associated with DKD stage, comprising 23 with positive and eight with negative normalized enrichment scores (Supplementary Table S3). Prominent positively associated programs included immune, interferon, interleukin, and extracellular-matrix processes, whereas oxidative phosphorylation, fatty acid metabolism, xenobiotic metabolism, and bile acid-related programs were negatively associated with increasing DKD stage. The Hallmark reactive oxygen species pathway was not significantly associated with DKD stage.

A complementary Reactome gene-set enrichment analysis was used to define the more detailed pathway landscape associated with DKD stage independently of the compound–target information. Of 1083 Reactome pathways initially evaluated, 129 representative pathways were retained after filtering and pathway collapsing for subsequent integration. Compound–target over-representation analysis was then performed independently across these 129 pathways using pathway-analysis target sets derived from the predefined target universes by retaining targets measurable in GSE142025 and excluding relationships classified primarily as direct compound handling/binding or handling/transport. This yielded 73 anserine targets and 83 RRR-α-tocopherol targets, including 11 shared targets. Sixteen pathways were significantly enriched for each compound after multiple-testing correction (Supplementary Table S3). Four pathways were significant for both compound–target sets in this primary analysis and were therefore classified as shared pathway-level convergence signals, leaving 12 significant compound-specific pathways for each compound. Among these, pathways containing at least one of the previously prioritized compound–DKD relationships were retained for focused integration, yielding six anserine-specific and six RRR-α-tocopherol-specific pathways. This focused retention step was applied only after the statistical pathway analyses and did not alter either the pathway-enrichment results or the previously established 21 compound–DKD relationships.

**Figure 4:**
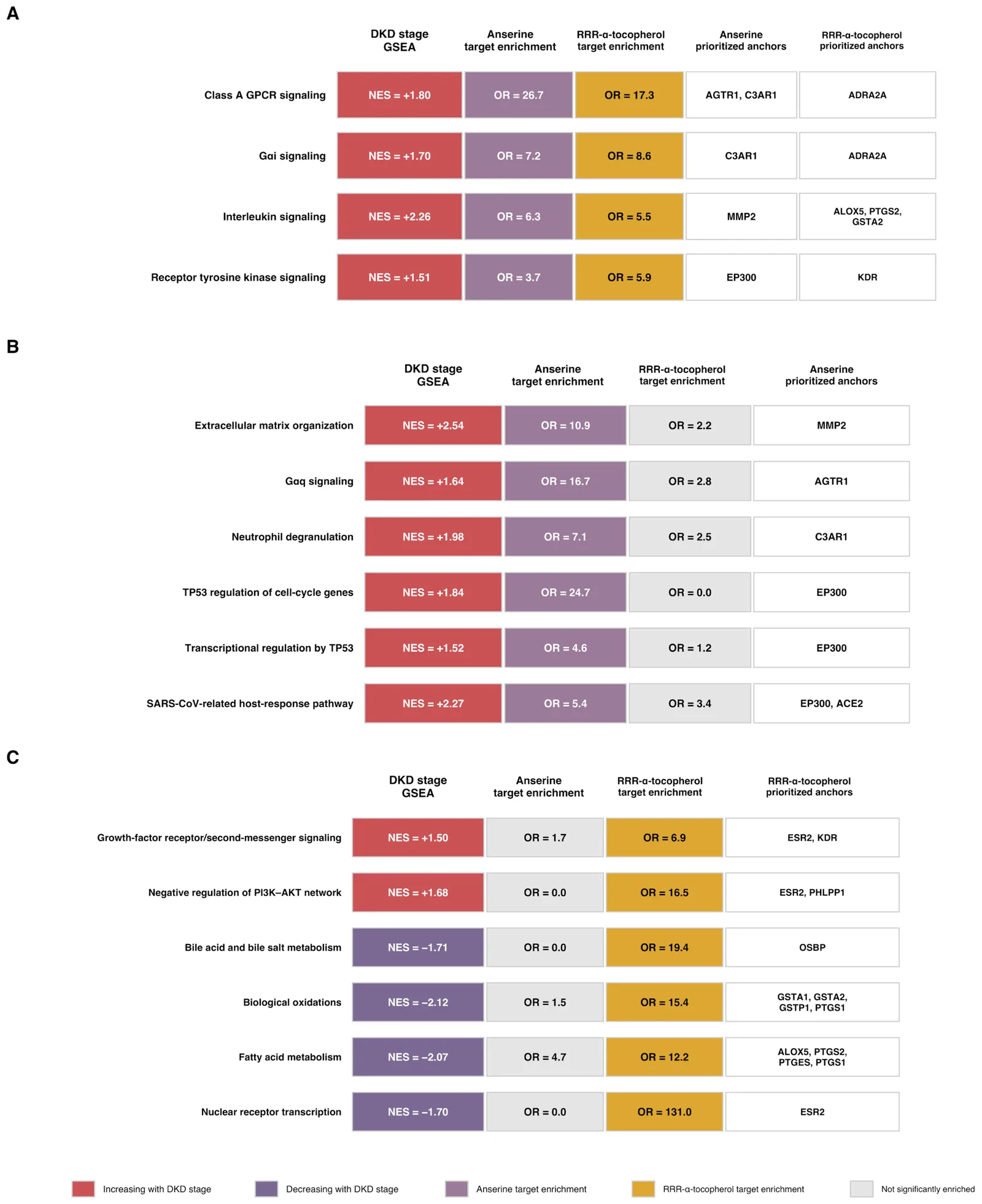
Integration of prioritized compound–diabetic kidney disease (DKD) relationships with shared and compound-specific Reactome pathways. (**A**) In the primary target-set analysis, four retained Reactome pathways were significantly enriched among both anserine and RRR-α-tocopherol target sets: Class A G protein-coupled receptor (GPCR) signaling, Gαi signaling, interleukin signaling, and receptor tyrosine kinase signaling. Disease-stage normalized enrichment scores (NES), compound–target-enrichment odds ratios (ORs), and prioritized compound–DKD genes represented within each pathway are shown. (**B**) Six pathways were significantly enriched among anserine targets but not RRR-α-tocopherol targets: extracellular-matrix organization, Gαq signaling, neutrophil degranulation, TP53 regulation of cell-cycle genes, transcriptional regulation by TP53, and a severe acute respiratory syndrome coronavirus (SARS-CoV)-related host-response pathway. The latter reflects generic host-response machinery and does not imply viral involvement. (**C**) Six pathways were significantly enriched among RRR-α-tocopherol targets but not anserine targets: growth-factor receptor and second-messenger signaling, negative regulation of the phosphoinositide 3-kinase (PI3K)-AKT network, bile acid and bile salt metabolism, biological oxidations, fatty acid metabolism, and nuclear-receptor transcription. Reactome over-representation analysis used one-sided Fisher exact tests with the 17,184 genes measurable in GSE142025 as background and Benjamini–Hochberg correction across 129 retained pathways. Red and purple disease-stage cells indicate positive and negative NES, respectively; mauve and gold cells indicate significant target enrichment for anserine and RRR-α-tocopherol; gray indicates non-significance. White cells containing gene symbols identify previously prioritized compound–DKD relationships represented within the corresponding pathways. Disease-stage direction reflects human DKD association and does not indicate compound-mediated activation, inhibition, restoration, or reversal.

In the primary target-set analysis, the four shared convergence pathways comprised Class A G protein-coupled receptor (GPCR) signaling, Gαi signaling, signaling by interleukins, and signaling by receptor tyrosine kinases, all of which were positively associated with increasing DKD stage (Figure 4A). Their classification as shared pathways was based on significant target enrichment for both compounds and was independent of the previously prioritized compound–DKD relationships. Subsequent mapping of those prioritized relationships onto the shared pathways showed that the pathway-level convergence was represented by different prioritized targets for the two compounds. Class A GPCR signaling was linked to AGTR1 and C3AR1 for anserine and ADRA2A for RRR-α-tocopherol, whereas Gαi signaling was linked to C3AR1 for anserine and ADRA2A for RRR-α-tocopherol. Interleukin signaling was associated with MMP2 for anserine and ALOX5, PTGS2, and GSTA2 for RRR-α-tocopherol, while receptor tyrosine kinase signaling was linked to EP300 for anserine and KDR for RRR-α-tocopherol.

The six retained anserine-specific pathways comprised extracellular-matrix organization, which contained the previously prioritized target MMP2; Gαq signaling, which contained AGTR1; neutrophil degranulation, which contained C3AR1; a severe acute respiratory syndrome coronavirus (SARS-CoV)-related host-response pathway, which contained EP300 and ACE2; TP53 regulation of cell-cycle genes, which contained EP300; and transcriptional regulation by TP53, which also contained EP300. All six pathways were positively associated with increasing DKD stage (Figure 4B). The SARS-CoV-related pathway designation reflects generic host-response machinery and should not be interpreted as evidence of viral involvement.

The six retained RRR-α-tocopherol-specific pathways comprised bile acid and bile salt metabolism, which contained the previously prioritized target OSBP; biological oxidations, which contained GSTA1, GSTA2, GSTP1, and PTGS1; growth-factor receptor and second-messenger signaling, which contained ESR2 and KDR; fatty acid metabolism, which contained ALOX5, PTGS2, PTGES, and PTGS1; negative regulation of the phosphoinositide 3-kinase (PI3K)-AKT network, which contained ESR2 and PHLPP1; and nuclear-receptor transcription, which contained ESR2. Growth-factor receptor and second-messenger signaling and negative regulation of the PI3K–AKT network were positively associated with increasing DKD stage, whereas bile acid and bile salt metabolism, biological oxidations, fatty acid metabolism, and nuclear-receptor transcription were negatively associated with increasing DKD stage (Figure 4C).

Collectively, the primary pathway analyses showed that the largely distinct anserine and RRR-α-tocopherol target profiles partially converged at the pathway level on four DKD stage-associated signaling pathways, while also retaining distinct compound-specific pathway associations. The prioritized compound–DKD relationships provided gene-level context for these pathway signals but did not determine the pathway-enrichment results. The robustness of the primary pathway architecture to a more conservative compound–target definition was evaluated separately in the target-confidence sensitivity analysis described below.

### 2.6. Renal Cellular Localization of Prioritized Compound–DKD Genes

**Figure 5:**
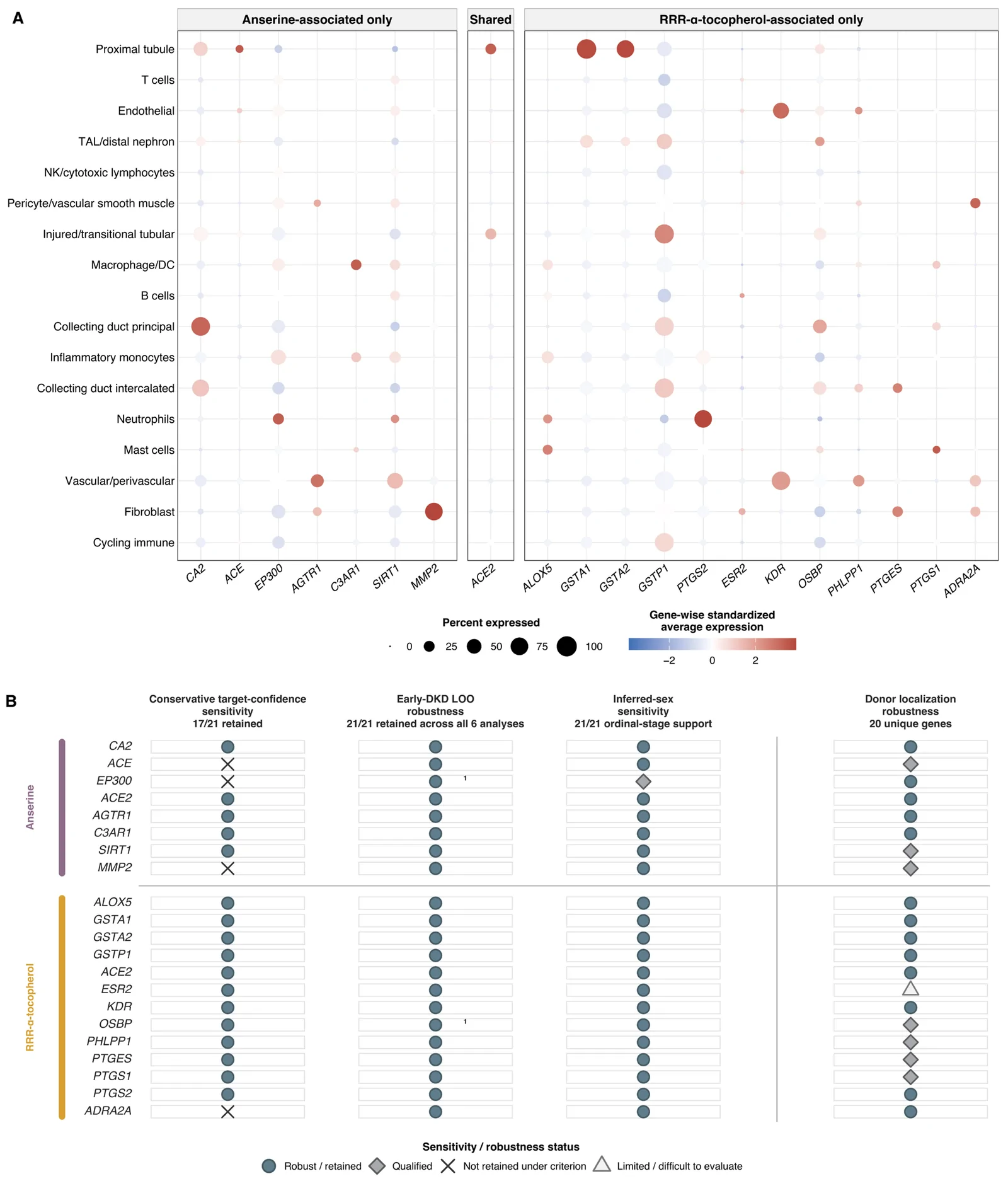
Renal cellular context and robustness of prioritized compound–diabetic kidney disease (DKD) relationships. (**A**) Single-cell localization of the 20 unique genes represented by the 21 prioritized compound–DKD relationships across 17 annotated renal and immune cell populations in GSE209781. Genes are grouped as anserine-associated only, shared, or RRR-α-tocopherol-associated only; *ACE2* is displayed once in the shared group but contributes one prioritized relationship to each compound. Dot size represents the percentage of cells expressing each gene within the corresponding population. Dot color represents gene-wise standardized average expression across the 17 populations and therefore supports within-gene comparisons of relative cellular localization only; color intensity should not be interpreted as an absolute expression comparison between different genes. The single-cell analysis is descriptive and does not establish cell-specific compound effects. (**B**) Sensitivity and robustness of the 21 previously prioritized compound–DKD relationships under conservative target-confidence restriction, leave-one-Early-DKD-out analysis, inferred-sex adjustment, and donor-level localization assessment. Status is analysis-specific and does not represent an overall relationship score or ranking. “Not retained under criterion” indicates that the relationship did not satisfy that specific sensitivity criterion and does not invalidate the original prioritized relationship; “Limited/difficult to evaluate” reflects insufficient evidence for confident evaluation rather than negative biological evidence. Under inferred-sex sensitivity, *EP300* retained significant ordinal-stage evidence, whereas the adjusted Advanced-DKD-versus-Control association was attenuated and was therefore classified as qualified. Superscript ^1^ indicates relationships for which monotonic classification changed in one of six leave-one-Early-DKD-out analyses, while the principal ordinal-stage and Advanced-DKD-versus-Control evidence remained significant and directionally concordant. Donor-localization robustness was evaluated at the unique-gene level (*n* = 20) using donor-resolved expression, donor cell-type coverage, six leave-one-donor-out reaggregations, and descriptive DKD-versus-non-DKD localization; consequently, the same *ACE2* localization status is shown for both compound-*ACE2* relationship rows.

To characterize the renal cellular context of the prioritized compound–DKD relationships, single-cell RNA-sequencing data from GSE209781 were analyzed across six donors. Following quality control, 48,314 cells were retained, and after exclusion of the mixed/doublet-like cluster, 48,148 cells distributed across 17 annotated renal and immune cell populations were used for localization analysis. The 21 prioritized compound–DKD relationships corresponded to 20 unique genes because *ACE2* was prioritized independently for both compounds. Localization of all 20 genes was evaluated descriptively using average normalized expression and the percentage of expressing cells across the 17 populations (Figure 5A; Supplementary Table S4).

The prioritized genes occupied diverse renal cellular contexts. Several showed predominantly tubular or epithelial localization, including *CA2* in collecting-duct populations, *ACE* and *ACE2* in proximal-tubular cells, *GSTA1* and *GSTA2* in the proximal tubule, and *GSTP1* in injured/transitional tubular populations. *OSBP* showed a predominantly thick ascending limb/distal-nephron pattern, whereas *PTGES* was represented across collecting-duct and stromal contexts. Immune-associated localization was observed for several genes, including *C3AR1* in macrophage/dendritic and other myeloid populations, *ALOX5* and *PTGS1* in immune populations including mast cells, and *PTGS2* prominently in neutrophils. *EP300* showed broader expression across renal cell populations, with neutrophils showing the highest average expression. Vascular or stromal localization characterized *AGTR1*, *KDR*, *PHLPP1*, and *ADRA2A*, while *MMP2* showed a prominent fibroblast-associated pattern. Other genes, including *SIRT1* and *ESR2*, showed comparatively heterogeneous or low-abundance localization patterns. These distributions provide descriptive renal cellular context for the prioritized relationships and do not imply cell-specific pharmacological action by either compound.

### 2.7. Sensitivity and Robustness of the Analytical Framework

Conservative restriction of compound–target evidence reduced the target sets to 26 anserine and 29 RRR-α-tocopherol targets, of which 19 and 25, respectively, were measurable in GSE142025. Under this more stringent definition, 12 of 19 anserine targets overlapped stage-associated genes (OR = 1.35, *p* = 0.347), whereas 20 of 25 RRR-α-tocopherol targets did so (OR = 3.15, *p* = 0.011). For monotonic stage-associated genes, the corresponding values were five of 19 for anserine (OR = 0.69, *p* = 0.828) and 13 of 25 for RRR-α-tocopherol (OR = 2.11, *p* = 0.048). At the relationship level, 17 of the 21 previously prioritized relationships remained target-eligible under the conservative criterion: all Tier 1 and Tier 3 relationships were retained, whereas the Tier 2 relationships *ACE* and *EP300* and the Tier 4 relationships *MMP2* and *ADRA2A* were not retained under this specific sensitivity criterion (Figure 5B; Supplementary Table S6). Reactome target-enrichment results were also more sensitive to target restriction for anserine: the number of significant pathways decreased from 16 to five for anserine and from 16 to seven for RRR-α-tocopherol. Of the four shared pathways identified in the primary analysis, receptor tyrosine kinase signaling was the only pathway that remained significantly enriched for both restricted target sets (Supplementary Table S3).

The GSE142025 disease-side results were highly stable to exclusion of individual Early DKD samples. Across the six leave-one-Early-DKD-out analyses, 9506–9650 genes remained significantly associated with ordinal DKD stage, with 98.62–99.06% of the original 9613 stage-associated genes retained in each analysis and all-gene stage-effect correlations ranging from 0.999806 to 0.999879. All 21 prioritized relationships retained significant ordinal-stage associations and significant Advanced-DKD-versus-Control differences with concordant directions in every leave-one-out analysis. Monotonic classification was unchanged in all six iterations for 19 of the 21 relationships; *EP300* and *OSBP* each changed monotonic classification in one iteration without loss of their principal ordinal-stage or Advanced-DKD-versus-Control evidence. Although exact membership of the stringent 66-gene signature was more threshold-sensitive, all 66 genes retained significant ordinal-stage associations and their original monotonic directions in every leave-one-out analysis (Figure 5B; Supplementary Table S6).

Adjustment for expression-inferred sex likewise supported the overall stability of the stage-associated transcriptional landscape. The adjusted ordinal model identified 9241 stage-associated genes, of which 9095 overlapped the primary 9613-gene set (Jaccard similarity = 0.932), with an all-gene stage-effect correlation of 0.990 and 100% direction concordance among genes significant in both models. All 21 prioritized relationships retained significant adjusted ordinal-stage associations with concordant directions. *EP300* showed greater pairwise sensitivity: its adjusted Advanced-DKD-versus-Control association no longer reached FDR < 0.05 (FDR = 0.0619), although its adjusted ordinal-stage association remained significant (FDR = 0.0472). In the corresponding target-enrichment sensitivity analysis, anserine stage enrichment was attenuated to borderline significance (49/77 targets; OR = 1.51, *p* = 0.051), whereas RRR-α-tocopherol enrichment remained significant (63/90 targets; OR = 2.01, *p* = 0.0012) (Figure 5B; Supplementary Table S6).

Relationship-level external disease replication provided an additional assessment of the human DKD evidence. Eight of the 21 relationships met the predefined replication criterion in GSE30528 and 14 in GSE96804; four replicated in both cohorts and 18 replicated in at least one cohort (Supplementary Table S6). *EP300* showed no demonstrated external replication, with one cohort unevaluable; *GSTP1* did not meet the replication criterion despite being evaluable in both cohorts; and *GSTA2* was not measurable in either external dataset and was therefore classified as unavailable rather than as a biological replication failure. Because external replication was already incorporated into the eligibility criteria for Tier 3 and Tier 4 relationships, these results should not be interpreted as an independent validation layer for those tiers. More broadly, external replication supports reproducibility of the corresponding gene–DKD associations and does not validate compound–target engagement.

Donor-aware analysis of GSE209781 further indicated that most principal renal-localization patterns were preserved after exclusion of individual donors (Figure 5B; Supplementary Table S6). Several genes showed highly reproducible localization across donor-resolved and leave-one-donor-out analyses, including *CA2*, *EP300*, *C3AR1*, *ACE2*, *GSTA1*, *GSTA2*, and *PTGS2*, while *AGTR1*, *ALOX5*, *GSTP1*, *KDR*, and *ADRA2A* retained stable broader cellular-compartment assignments despite some subtype variation. Other localization patterns were supported but more heterogeneous across donors or conditions, including *ACE*, *SIRT1*, *OSBP*, *PHLPP1*, *PTGES*, and *PTGS1*. The fibroblast localization of *MMP2* remained evident after donor exclusion but was coverage-limited because fibroblasts were absent in two donors, whereas *ESR2* localization was difficult to evaluate confidently because of low expression and donor dependence. These analyses assess localization stability only and do not establish cell-type-specific DKD differential expression or compound action.

## 3. Discussion

This study used an integrative human disease-centered framework to examine the molecular relationships of anserine and RRR-α-tocopherol with DKD while maintaining compound–target evidence and human disease evidence as distinct analytical dimensions. Integration of independently assembled compound–target profiles with stage-resolved human kidney transcriptomics prioritized 21 compound–DKD relationships involving 20 unique genes: eight relationships for anserine and 13 for RRR-α-tocopherol. These relationships constituted the terminal gene-level result and were established before pathway and single-cell analyses. The two compounds showed limited overlap in their predefined target profiles, yet both target sets intersected the DKD-associated transcriptional landscape and subsequently showed partial convergence at the pathway level. Thus, the principal finding is not a small set of shared molecular targets, but rather a pattern in which largely distinct compound-associated molecular profiles intersect human DKD through different gene-level relationships while converging on selected disease-associated biological programs.

The human transcriptomic analysis provided the disease-centered foundation for this integration. GSE142025 showed extensive transcriptional remodeling across Control, Early DKD, and Advanced DKD, with the greatest differences observed in Advanced DKD. Of 9613 genes associated with the ordinal disease-stage variable, 5841 additionally showed monotonic expression across the three groups, while a stringent 66-gene signature captured changes that were directionally consistent across both adjacent-stage comparisons. Because these data are cross-sectional, these patterns represent associations with increasing DKD stage rather than longitudinal disease progression. Nevertheless, their broad stability in leave-one-Early-DKD-out and inferred-sex sensitivity analyses, together with partial replication in two independent glomerular DKD cohorts, supports the presence of a reproducible disease-associated transcriptional framework against which the compound–target profiles could be evaluated.

Within this framework, both predefined compound–target sets were enriched among genes associated with DKD stage, although their enrichment patterns differed. RRR-α-tocopherol targets were additionally enriched among genes showing monotonic expression across the three disease groups, whereas anserine targets were not. This difference should not be interpreted as evidence of greater biological activity or therapeutic potential for RRR-α-tocopherol, because the analyses were not designed as a formal comparison of the two compounds. Rather, together with the limited overlap between their predefined target sets, it indicates that anserine and RRR-α-tocopherol intersect different components of the DKD-associated molecular landscape. The subsequent pathway and renal single-cell analyses therefore provide complementary biological context for the 21 prioritized relationships rather than additional criteria for selecting or ranking them.

### 3.1. Human DKD Transcriptional Remodeling and External Validation

The stage-resolved transcriptional landscape was consistent with the increasing biological complexity of established DKD. Fan et al. originally reported distinct transcriptional patterns between early and advanced diabetic nephropathy, including greater representation of immune- and fibrosis-related genes in advanced disease [5]. The present reanalysis extends that observation by applying an ordinal framework across Control, Early DKD, and Advanced DKD and by distinguishing genes associated with disease stage from those additionally showing monotonic expression across the three groups. The accompanying pathway profile, characterized by increasing inflammatory, interleukin, immune, and extracellular-matrix programs together with decreasing representation of several metabolic programs, is consistent with current models of DKD in which metabolic and hemodynamic disturbances become increasingly intertwined with inflammatory, immune, and fibrotic mechanisms [13]. Independent transcriptomic analysis of microdissected human diabetic kidney tissue has similarly identified prominent inflammatory and extracellular-matrix signals in glomeruli, together with immune- and injury-related alterations in the tubulointerstitium [14]. These concordant observations provide biological context for interpreting the compound-associated relationships against a disease landscape involving coordinated inflammatory, structural, and metabolic remodeling rather than isolated molecular abnormalities.

External assessment further supported the disease-associated component of this framework, while also illustrating the expected heterogeneity of human DKD transcriptomic datasets. GSE30528 and GSE96804 were independently generated from human glomerular tissue using different platforms and cohort designs [15,16]. Genome-wide effect-size concordance with the GSE142025 Advanced-DKD-versus-Control contrast was modest, and replication of the stringent 66-gene signature was incomplete; however, substantial directional concordance was observed among genes significant in both discovery and external analyses, and subsets of the stringent signature met the predefined replication criterion in each cohort. Differences in cohort composition, tissue sampling, platform coverage, and analytical sensitivity likely contribute to the incomplete overlap. Accordingly, external replication should be interpreted as supporting reproducibility of specific gene–DKD associations rather than as evidence for compound–target engagement. This distinction becomes particularly important when interpreting the prioritized compound–DKD relationships, because compound–target evidence and disease evidence were deliberately maintained as separate dimensions throughout the study.

### 3.2. Anserine-Associated Molecular Context

The eight prioritized anserine–DKD relationships place anserine within several biological contexts relevant to diabetic kidney injury, while differing substantially in the strength and nature of their compound–target evidence. Experimental work has shown that anserine can influence renal stress responses under diabetic conditions: in high-glucose-exposed human HK-2 cells, anserine increased heat shock protein 70 and heme oxygenase-1 expression and reduced protein carbonylation, whereas short-term administration to diabetic db/db mice reduced proteinuria and vascular permeability and improved glycemic measures [8]. These observations provide biological plausibility for considering anserine in diabetic kidney injury but do not identify the molecular mediators responsible for those effects. The present analysis therefore contributes a complementary human disease-centered framework in which the eight prioritized relationships are interpreted according to their individual compound–target evidence, DKD association, and renal biological context rather than as a smaller set of privileged molecular endpoints.

Among these relationships, *CA2* is notable because the compound–target connection is supported by direct human biochemical evidence. Anserine has been shown to activate human carbonic anhydrase II, together with several other carbonic anhydrase isoforms, in the micromolar concentration range [17]. In the present study, *CA2* was also associated with DKD stage and localized predominantly to tubular and collecting-duct populations, providing a coherent renal context for this experimentally demonstrated interaction. This convergence does not establish that modulation of *CA2* mediates the renal effects previously reported for anserine, but it makes the anserine–*CA2* relationship a particularly tractable hypothesis for direct experimental testing.

Other prioritized relationships extend the anserine-associated profile into vascular, immune, and matrix-remodeling biology. *AGTR1* provides clinically relevant disease context because angiotensin II type 1 receptor signaling is an established component of diabetic kidney disease; in the RENAAL trial, losartan reduced important renal outcomes in patients with type 2 diabetes and nephropathy [18]. Its vascular/perivascular localization in the present analysis is therefore biologically consistent with its established renal hemodynamic and vascular role. However, the anserine–*AGTR1* relationship is supported here by computational compound–target evidence integrated with human DKD evidence and should not be interpreted as evidence that anserine acts as an angiotensin II type 1 receptor antagonist. Similarly, *C3AR1* connects the anserine profile to complement-associated inflammation. Increased renal C3a-receptor expression has been reported in human diabetic nephropathy, and C3a-receptor deficiency attenuated albuminuria and renal pathological injury in diabetic mice [19]. The myeloid localization of *C3AR1* observed here is consistent with the immune and interleukin-associated disease programs identified in the stage-resolved analyses, while remaining insufficient to infer direct modulation of C3a receptor signaling by anserine.

*MMP2* illustrates both the biological relevance and the evidentiary uncertainty within the anserine profile. Human and experimental studies have reported apparently divergent patterns of matrix metalloproteinase-2 (MMP-2) biology in diabetic kidney disease, including reduced glomerular *MMP2* expression [20], worsening albuminuria and matrix accumulation after genetic deletion of *Mmp2* in diabetic mice [21], and increased active MMP-2 in diabetic kidney tissue [22]. These findings suggest that MMP-2 function is likely to depend on disease stage, renal compartment, and biological context. In the present analysis, *MMP2* showed fibroblast-associated localization, but that localization was coverage-limited across donors, and the anserine–*MMP2* relationship was not retained under the more conservative compound–target confidence definition. It is therefore better viewed as a contextually plausible but comparatively uncertain relationship rather than as a dominant mechanistic explanation for anserine in DKD.

Taken together, the anserine profile spans tubular biochemical regulation, renin–angiotensin signaling, complement-associated inflammation, matrix remodeling, and additional disease-associated relationships represented by *ACE*, *EP300*, *SIRT1*, and *ACE2*. Importantly, this breadth should not be interpreted as equivalent confidence across all eight relationships. Under the conservative compound–target sensitivity analysis, five of the eight relationships remained target-eligible, whereas *ACE*, *EP300*, and *MMP2* did not. This pattern indicates that the disease-side evidence for anserine remains biologically informative while some compound–target assignments are more sensitive to the stringency of the target definition. The appropriate interpretation is therefore a heterogeneous set of experimentally testable anserine–DKD relationships, rather than a single validated mechanism or a reduced subset of definitive targets.

### 3.3. RRR-α-Tocopherol-Associated Molecular Context

The 13 prioritized RRR-α-tocopherol–DKD relationships span inflammatory, oxidative-metabolic, tubular, and vascular biological contexts, with several supported by direct human-experimental evidence and others by computational compound–target prediction. Clinical data provide relevant, although not compound-specific, context. In a randomized, double-blind, placebo-controlled trial in patients with diabetic nephropathy, high-dose vitamin E supplementation reduced urinary protein and the protein-to-creatinine ratio and was accompanied by reductions in several inflammatory and oxidative-stress markers [11]. Because that study did not establish the same stereochemically defined RRR-α-tocopherol entity evaluated here, it should be regarded as broader translational context for vitamin E rather than direct validation of the present RRR-α-tocopherol relationships.

Among the prioritized relationships, *ALOX5* provides a particularly coherent connection between direct compound-related evidence and DKD-associated inflammatory biology. In activated human monocytes, α-tocopherol reduced tumor necrosis factor-α (TNF-α) production through a mechanism linked to inhibition of the 5-lipoxygenase pathway, and the experimental work included RRR-α-tocopherol exposure [23]. In the present analysis, *ALOX5* increased with DKD stage, localized predominantly to immune/myeloid populations, and mapped to both interleukin signaling and fatty acid metabolism. These convergent observations support the biological plausibility of the RRR-α-tocopherol–*ALOX5* relationship but do not establish that renal ALOX5 inhibition mediates the therapeutic effect of RRR-α-tocopherol in DKD.

*GSTA1* and *GSTA2* provide a complementary proximal-tubular and oxidative-metabolic context. RRR-α-tocopherol has been shown to inhibit purified human glutathione S-transferase (GST) A1-1 and GST A2-2 in a concentration-dependent manner, with inhibition also observed in human liver cytosol at higher concentrations [24]. In the present DKD analysis, both genes decreased with increasing disease stage, showed strong proximal-tubular localization, and were represented within the negatively stage-associated biological oxidations pathway. This alignment places directly supported RRR-α-tocopherol–GST interactions within a renal metabolic compartment affected in DKD, but it does not imply that RRR-α-tocopherol restores GST expression or reverses the declining disease-associated pathway signal. The direction of the biochemical interaction and the direction of disease-associated transcript abundance should therefore remain analytically distinct.

*KDR* extends the RRR-α-tocopherol profile into vascular and endothelial biology. Unlike *ALOX5*, *GSTA1*, and *GSTA2*, the RRR-α-tocopherol–*KDR* relationship is based on computational compound–target evidence rather than direct experimental interaction. Its disease relevance is nevertheless supported by human diabetic kidney observations. Hohenstein et al. reported that vascular endothelial growth factor (VEGF) receptor activity in the glomerular endothelium varied with the severity of diabetic glomerular injury, with increased activation in mildly affected glomeruli and reduced activation together with capillary rarefaction in more-advanced lesions [25]. In the present study, *KDR* decreased monotonically with increasing DKD stage, met the external-replication criterion in both independent cohorts, and localized predominantly to endothelial and vascular/perivascular populations. These concordant disease-side observations strengthen the renal vascular context of *KDR*, while leaving the direct pharmacological relationship with RRR-α-tocopherol predictive.

The broader RRR-α-tocopherol profile extends beyond these illustrative relationships and includes *ACE2*, *PTGS2*, *ESR2*, *OSBP*, *PHLPP1*, *PTGES*, *PTGS1*, *GSTP1*, and *ADRA2A*, thereby spanning inflammatory, lipid-related, receptor-signaling, metabolic, and vascular processes. Importantly, the conservative target-confidence analysis retained 12 of the 13 prioritized RRR-α-tocopherol relationships, with only *ADRA2A* falling outside the restricted target definition. This relative stability indicates that most of the RRR-α-tocopherol compound–target assignments were robust to greater target-evidence stringency, but it should not be interpreted as evidence that RRR-α-tocopherol is more effective or more biologically important than anserine. Rather, it reflects differences in the evidentiary composition of the two compound–target profiles. The 13 relationships are therefore best interpreted as a heterogeneous but largely target-confidence-robust set of experimentally testable molecular hypotheses.

### 3.4. Pathway Convergence, Complementarity, and Renal Cellular Context

Despite the limited overlap between the predefined compound–target sets, the primary pathway analysis identified four Reactome pathways significantly enriched for both anserine and RRR-α-tocopherol targets: Class A G protein-coupled receptor signaling, Gαi signaling, interleukin signaling, and receptor tyrosine kinase signaling. All four pathways were positively associated with increasing DKD stage in the independent disease-side GSEA. This pattern is consistent with the broader view of DKD as a disorder involving interacting hemodynamic, inflammatory, metabolic, vascular, and fibrotic systems rather than disruption of a single pathway [2]. Importantly, however, disease-stage enrichment and compound–target over-representation represent distinct analytical dimensions: the positive DKD-stage normalized enrichment scores do not indicate that either compound activates these pathways, and shared target enrichment does not establish a common pharmacological mechanism. Rather, the primary analysis supports partial pathway-level convergence of otherwise largely distinct compound–target profiles.

The prioritized relationships mapped to these shared pathways through different proximal genes, further emphasizing this distinction. For example, Class A GPCR and Gαi signaling included *AGTR1* and *C3AR1* on the anserine side and *ADRA2A* on the RRR-α-tocopherol side; interleukin signaling included *MMP2* for anserine and *ALOX5*, *PTGS2*, and *GSTA2* for RRR-α-tocopherol; and receptor tyrosine kinase signaling included *EP300* and *KDR*, respectively. The compound-specific pathway profiles also differed, with anserine-associated signals including extracellular-matrix, Gαq, neutrophil, and TP53-related programs, whereas RRR-α-tocopherol targets additionally intersected biological oxidations, fatty acid metabolism, bile acid-related metabolism, nuclear-receptor transcription, and PI3K-AKT-related signaling. These patterns describe where the independently defined target sets intersect the DKD-associated pathway landscape; they should not be interpreted as evidence that either compound activates stage-increasing pathways or restores those that decline with increasing disease stage.

The conservative compound–target sensitivity analysis further qualified the pathway interpretation. Under the more restrictive target definition, the primary four-pathway shared architecture contracted to a single shared significant pathway, receptor tyrosine kinase signaling. None of the six focused anserine-associated pathways remained significant under this stricter definition, whereas five of the six focused RRR-α-tocopherol-associated pathways were retained. This sensitivity does not invalidate the primary pathway results, which were generated from the prespecified primary target sets, but it indicates that several pathway associations, particularly within the anserine profile, depend on compound–target relationships with greater evidentiary uncertainty. Receptor tyrosine kinase signaling therefore represents the most target-confidence-robust shared pathway signal, while the remaining primary convergence pathways should be interpreted as biologically plausible but more target-definition-sensitive observations.

Previous experimental work in streptozotocin-induced diabetic rats reported effects of anserine and α-tocopherol, individually and in combination, on glycemic indices and markers of oxidative stress, inflammation, and advanced glycation end products [12]. Although these findings provide useful experimental context for considering the two compounds together, they do not establish that the pathway convergence observed here reflects pharmacological synergy or the mechanism underlying those treatment-level effects.

Renal single-cell analysis provided a separate cellular layer of context for the 20 unique prioritized genes. Human single-cell and single-nucleus studies have demonstrated substantial cell-type heterogeneity in DKD, including alterations involving tubular, endothelial, stromal, and immune populations [3,4]. Consistent with this complexity, the prioritized genes in the present study were distributed across all 17 retained renal and immune cell populations rather than concentrating within a single compartment. Representative patterns included tubular or collecting-duct localization for *CA2*, proximal-tubular localization for *GSTA1*, *GSTA2*, and *GSTP1*, immune/myeloid localization for *C3AR1* and *ALOX5*, vascular or endothelial localization for *AGTR1*, *KDR*, and *ADRA2A*, and fibroblast-associated expression of *MMP2*. *EP300* showed broader expression across renal populations, with neutrophils showing the highest average expression in the donor-resolved analyses. These patterns provide spatial biological context for the prioritized relationships but do not demonstrate cell-type-specific compound action or DKD differential expression.

Donor-aware analyses supported the stability of many of these broad localization patterns, particularly for genes such as *CA2*, *C3AR1*, *ACE2*, *GSTA1*, *GSTA2*, *PTGS2*, *ALOX5*, *KDR*, and *ADRA2A*, while other genes showed greater donor or condition dependence. The fibroblast-associated localization of *MMP2* was coverage-limited because fibroblasts were absent or sparsely represented in some donors, and *ESR2* was difficult to evaluate confidently because of low expression and donor dependence. Thus, the single-cell analysis strengthens the anatomical interpretability of the 20-gene set while preserving appropriate uncertainty for sparsely represented or heterogeneous signals. Pathway convergence and cellular localization should therefore be viewed as complementary contextual dimensions surrounding the 21 prioritized relationships, not as additional selection criteria or evidence of compound efficacy.

### 3.5. Translational Implications, Strengths, and Limitations

The present findings should be interpreted within the broader therapeutic complexity of DKD. Contemporary management combines interventions directed at complementary physiological and molecular processes, including renin–angiotensin system blockade, sodium–glucose cotransporter 2 inhibitors, glucagon-like peptide-1 receptor agonists, and nonsteroidal mineralocorticoid receptor antagonists, yet substantial residual kidney and cardiovascular risk persists [1]. The vascular context identified for several prioritized genes is also relevant to the broader cardiorenal burden of chronic kidney disease(CKD). CKD is associated with a higher prevalence of peripheral artery disease, particularly with increasing CKD severity, and with poorer outcomes following peripheral revascularization [26]. These clinical associations reinforce the importance of vascular disease within the wider kidney–cardiovascular continuum but do not establish a DKD-specific molecular mechanism or implicate either anserine or RRR-α-tocopherol in peripheral vascular disease. More broadly, the molecular heterogeneity observed here is consistent with the view that DKD cannot be adequately represented by a single pathogenic pathway or cellular compartment.

The present analysis does not establish anserine or RRR-α-tocopherol as therapeutic interventions for DKD, nor does pathway convergence imply benefit from combined administration. Rather, the 21 prioritized compound–DKD relationships define a set of experimentally testable hypotheses whose evidentiary foundations differ across compound–target support, human DKD association, external replication, pathway context, and renal localization. Experimental follow-up should therefore be relationship-specific. Direct target-engagement studies would be particularly informative for computationally predicted relationships, whereas relationships already supported by human biochemical evidence could be examined for consequences in renal cell systems and diabetic injury models relevant to their observed localization. Such studies should distinguish target engagement from downstream functional effects and should evaluate concentration dependence, tissue exposure, and biological directionality rather than assuming that association with a disease-altered gene or pathway predicts therapeutic normalization.

Several design features strengthen the study. Compound–target evidence was assembled independently of the DKD analyses, reducing circularity and ensuring that disease-associated findings did not determine the initial target universes. The 21 prioritized relationships were established before pathway and single-cell analyses, so these downstream analyses contextualized rather than generated the terminal gene-level result. The disease component was anchored in human kidney transcriptomic data spanning Control, Early DKD, and Advanced DKD, with complementary assessment in two independent glomerular cohorts. Integration of bulk transcriptomics, pathway analysis, and human renal single-cell localization allowed the prioritized relationships to be examined across multiple levels of biological organization. Finally, maintaining compound–target evidence, disease association, external replication, pathway context, and cellular localization as separate evidence dimensions avoided collapsing heterogeneous forms of support into an opaque composite score or allowing supporting analyses to create a second prioritization endpoint.

The study also has important limitations. It is computational and does not provide new experimental confirmation that the prioritized targets mediate biological effects of either compound in DKD. Compound–target evidence was heterogeneous, ranging from direct human biochemical or functional evidence to computational prediction and translationally limited experimental support. This heterogeneity was reflected in the conservative target-confidence analysis, in which 17 of the 21 prioritized relationships remained target-eligible under the more restrictive definition. The discovery cohort was relatively small and unbalanced, particularly the Early DKD group (*n* = 6), and was cross-sectional; ordinal associations therefore describe differences across disease stages rather than longitudinal progression. Although leave-one-Early-DKD-out analyses showed strong stability of the discovery-stage landscape and all 21 prioritized relationships retained their core disease-side evidence, these analyses cannot eliminate uncertainty associated with the limited Early DKD sample size. Clinical sex was not reliably available at the individual-sample level and therefore required an expression-inferred sensitivity analysis rather than direct covariate adjustment using documented sex. That analysis broadly supported the primary findings, although the pairwise Advanced-DKD-versus-Control association for *EP300* was attenuated after adjustment while its ordinal-stage association remained significant.

External replication should likewise be interpreted with appropriate restraint. Eighteen of the 21 prioritized relationships met the predefined gene–DKD replication criterion in at least one external cohort, but genome-wide effect-size concordance between the discovery and external datasets was only modest. Incomplete replication is plausible given differences in cohort composition, disease severity, transcriptomic platform, tissue sampling, and gene coverage. Moreover, external-replication tests reproducibility of gene–DKD associations and does not validate compound–target engagement. Lack of demonstrated replication was also not equivalent across relationships: some genes were evaluable but did not meet the criterion, whereas others were incompletely measurable. These distinctions are important because the external cohorts provide supporting disease evidence rather than a new validation-based ranking of the 21 relationships.

The renal single-cell analysis was also descriptive and subject to sampling constraints. GSE209781 comprised only six donors, and the analysis was designed to characterize cellular localization rather than infer cell-type-specific DKD differential expression. A confidently identifiable podocyte population was not recovered, preventing assessment within this key DKD cell type. Donor-aware analyses supported many broad localization patterns but also showed greater uncertainty for sparsely represented or donor-dependent signals, including *MMP2* and *ESR2*. More fundamentally, expression of a prioritized gene in a renal cell population does not establish that either compound reaches that compartment at pharmacologically relevant concentrations, engages the corresponding protein, or modifies its activity in vivo. The present framework likewise does not address pharmacokinetics, metabolism, tissue exposure, dose–response relationships, safety, or pharmacodynamic interactions between anserine and RRR-α-tocopherol.

Taken together, this study defines 21 prioritized compound–DKD relationships involving 20 unique genes by integrating independently established compound–target evidence with stage-resolved human DKD transcriptomics. The largely distinct anserine and RRR-α-tocopherol target profiles showed partial pathway-level convergence while retaining different gene-level, pathway, and renal cellular contexts. Sensitivity and robustness analyses strengthened several components of this framework while also identifying relationships and pathway signals that were more dependent on target definition, cohort composition, or cellular sampling. These findings therefore provide a transparent, human disease-centered and hypothesis-generating framework for subsequent experimental testing, rather than evidence of therapeutic efficacy, a confirmed mechanism of action, or a requirement for combined administration of the two compounds.

## 4. Materials and Methods

### 4.1. Study Design and Analytical Framework

This study used an integrative computational framework to investigate the molecular relationships of anserine and RRR-α-tocopherol with human DKD (Figure 1). Compound–target evidence and human DKD evidence were established independently before integration. For each compound, target relationships were assembled from the primary literature, curated public databases, and computational target-prediction resources to define predefined compound-specific target sets. In parallel, stage-associated transcriptional changes were characterized in GSE142025 across Control, Early DKD, and Advanced DKD, with GSE30528 and GSE96804 subsequently used to assess replication of gene–DKD associations.

The predefined compound–target sets were then integrated separately with the GSE142025 transcriptional results to assess target enrichment within the stage-associated DKD landscape and to prioritize compound–DKD relationships using the predefined four-tier evidence framework. Compound–target evidence and human disease evidence were maintained as distinct evidence dimensions throughout this process, and external replication was interpreted as support for the corresponding gene–DKD association rather than validation of compound–target engagement. This integration yielded 21 prioritized compound–DKD relationships involving 20 unique genes, with *ACE2* represented independently for both compounds. These relationships constituted the terminal gene-level result and were established before downstream pathway and single-cell analyses.

Pathway context was evaluated as a separate downstream analytical branch. DKD stage-associated pathway patterns were characterized by gene-set enrichment analysis, whereas compound–target Reactome over-representation analysis was performed independently using the pathway-analysis target sets. The prioritized compound–DKD relationships were subsequently mapped onto the resulting pathway signals for interpretation, but did not determine the pathway-enrichment results. In a separate downstream branch, the 20 unique prioritized genes were localized descriptively across annotated renal and immune cell populations in GSE209781.

Component-specific sensitivity and robustness analyses were additionally performed to assess target-confidence restriction, discovery-cohort stability, inferred-sex adjustment, external disease-replication support, and donor-level stability of renal localization. These analyses were used to evaluate the robustness of the corresponding analytical components and did not introduce a further prioritization step or redefine the 21 prioritized relationships.

### 4.2. Compound–Target Evidence Collection and Curation

#### 4.2.1. Compound Identity and Evidence Sources

Anserine and RRR-α-tocopherol were curated as chemically defined entities using PubChem [27] and ChEMBL [28] identifiers. Anserine corresponded to PubChem compound identifier (CID) 112072 and ChEMBL CHEMBL448301, whereas RRR-α-tocopherol corresponded to PubChem CID 14985 and ChEMBL CHEMBL47. The corresponding predefined target universes ultimately comprised 100 targets for anserine and 111 targets for RRR-α-tocopherol.

Compound–target evidence was assembled independently for each compound from complementary sources, including the primary literature, curated public databases, and computational target-prediction resources. Separate source-specific records were maintained for experimental evidence, curated-database evidence, SwissTargetPrediction [29], similarity ensemble approach (SEA) predictions [30], and compound handling- or transport-related evidence. These source-specific evidence streams, together with the integrated master evidence table, curation rules, references, search audit, and final target-group assignments, are provided in Supplementary Data S1.

#### 4.2.2. Evidence Classification and Curation

Evidence from the individual source-specific datasets was integrated at the target level into a master evidence table. When the same target was supported by more than one source, it was represented once in the compound-specific target set while retaining the applicable evidence classes and source provenance. Thus, experimental, curated-database, computational-prediction, and compound-handling evidence could coexist for an individual target without being counted as independent target entries.

Compound–target relationships were classified according to the nature and strength of the supporting evidence. Experimental relationships were distinguished as direct human biochemical evidence, human functional experimental evidence, or supportive direct evidence obtained in non-human or otherwise translationally limited settings. Computational predictions were retained as a separate evidence class and were not interpreted as experimental validation. Relationships primarily reflecting compound binding, transport, handling, or metabolism were annotated separately from candidate mechanistic target relationships. Species attribution, stereochemical correspondence, experimental context, and other limitations were retained during curation rather than used to upgrade the evidentiary classification of a relationship. The primary-literature records underlying these classifications–including retained direct or functional experimental evidence [17,23,24,31,32,33,34], compound-handling or transport evidence [35,36,37,38,39,40,41,42,43], and supportive, held, or excluded records retained during evidence adjudication [44,45,46,47,48,49,50,51,52]–are documented with their target-level provenance in Supplementary Data S1.

Where multiple evidence types supported the same target, the stronger evidence did not erase the provenance or limitations of the weaker evidence, and the presence of multiple sources did not increase the number of targets in the compound-specific universe. The resulting evidence architecture therefore preserved both nonredundant target identity and the qualitative distinctions among experimental, computational, and handling-related support.

#### 4.2.3. Definition of the Predefined Compound–Target Sets

The consolidated master evidence table was used to define nonredundant predefined target sets for each compound before integration with any DKD transcriptomic, pathway, or single-cell results. The final sets comprised 100 unique targets for anserine and 111 unique targets for RRR-α-tocopherol. Their intersection contained 16 shared targets, corresponding to 84 anserine-specific and 95 RRR-α-tocopherol-specific targets and a union of 195 unique targets overall.

These target sets were established independently of the subsequent human DKD analyses and were not altered on the basis of disease-associated transcription, pathway enrichment, or renal cell-type localization. This separation ensured that subsequent compound–DKD integration evaluated predefined compound–target evidence against the human disease data rather than allowing disease-related results to determine the initial target universes. The complete target-level evidence provenance and source-specific supporting records are provided in Supplementary Data S1.

#### 4.2.4. Conservative Target-Confidence Sensitivity Analysis

To evaluate the dependence of downstream findings on compound–target evidence confidence, a conservative sensitivity analysis was performed using a more restrictive target definition established independently of the DKD transcriptomic, external-replication, pathway, and single-cell results. Relationships supported by direct human biochemical evidence or human functional experimental evidence and retained as candidate target relationships were included in the restricted experimental component.

For computational-only relationships, prediction strength was ranked separately within each compound. Within-compound prediction percentiles were calculated as the average rank of the prediction divided by the total number of computational-only targets for that compound, with average ranks used for tied values. Computational-only targets with a prediction percentile ≥ 0.75 were retained in the restricted set. Relationships supported only by non-human direct experimental evidence, compound handling or transport, uncertain assignments, stereochemical mismatch, or computational predictions below this threshold were excluded from the restricted sensitivity set.

The 0.75 prediction-percentile threshold was used solely for this conservative sensitivity analysis and was not part of the criteria used to construct the primary predefined target sets. The restricted target sets were subsequently used to reassess compound–target enrichment within the stage-associated DKD transcriptomic landscape, the target-confidence status of the 21 previously prioritized compound–DKD relationships, and Reactome compound–target over-representation. These analyses were used to evaluate sensitivity to target-definition stringency and did not replace or redefine the primary compound–target sets or the previously established prioritization framework.

### 4.3. Human DKD Transcriptomic Discovery Analysis

#### 4.3.1. Dataset Acquisition and Expression-Matrix Preparation

The human DKD transcriptomic dataset GSE142025, generated by Fan et al. [5], was obtained from the Gene Expression Omnibus (GEO). The deposited dataset comprised 36 kidney biopsy samples available for analysis: nine Controls, six Early DKD samples, and 21 Advanced DKD samples. The original publication described 22 Advanced DKD samples, whereas 21 were represented in the deposited GEO series used here. The study was therefore treated as a cross-sectional stage-resolved dataset rather than a longitudinal cohort.

Processed sample-level expression files were extracted from the GSE142025 GEO archive and assembled into a common gene-by-sample matrix. Each deposited sample file contained 17,184 gene symbols, and gene order was confirmed to be identical across all 36 files. No duplicated gene symbols or missing expression values were present. The resulting matrix therefore contained 17,184 genes across 36 samples. The deposited expression values were continuous and approximately log2-scaled and were used without additional transformation or between-sample normalization.

#### 4.3.2. Principal Component Analysis

Global transcriptomic structure was assessed by principal component analysis (PCA). Gene-wise variance was calculated across all 36 samples, and the 1000 genes with the highest variance were selected. PCA was performed on the transposed expression matrix of these genes using centered, unscaled expression values (center = TRUE, scale. = FALSE). The proportion of variance explained by each principal component was calculated from the corresponding eigenvalues.

#### 4.3.3. Differential-Expression Analysis

Differential expression was evaluated using the limma framework [53]. Samples were represented as a three-level categorical factor corresponding to Control, Early DKD, and Advanced DKD, and a no-intercept design matrix was fitted to the full 17,184-gene expression matrix. Three prespecified pairwise contrasts were evaluated: Early DKD versus Control, Advanced DKD versus Control, and Advanced DKD versus Early DKD. Contrast-specific statistics were obtained after empirical Bayes moderation using eBayes. *p* values were adjusted for multiple testing using the Benjamini–Hochberg procedure [54], with a false discovery rate (FDR) < 0.05 defining statistical significance. No additional absolute log-fold-change threshold was imposed.

#### 4.3.4. Ordinal-Stage-Association and Stringent Stage-Associated Gene Definition

To identify transcriptional changes associated with increasing DKD stage, disease groups were additionally encoded as an ordinal variable with Control = 0, Early DKD = 1, and Advanced DKD = 2. For each gene, a linear model containing an intercept and the ordinal-stage variable was fitted using limma, followed by empirical Bayes moderation. Benjamini–Hochberg FDR < 0.05 was used to define significant stage-associated genes. Because the samples were cross-sectional, the resulting coefficient was interpreted as an association with increasing DKD stage rather than as a longitudinal progression effect.

For each gene, mean expression was calculated separately in the Control, Early DKD, and Advanced DKD groups. A gene was classified as monotonically increasing when its group means satisfied Control < Early DKD < Advanced DKD, and as monotonically decreasing when Control > Early DKD > Advanced DKD. Monotonic stage-associated genes were those meeting one of these directional criteria together with an ordinal-stage FDR < 0.05.

A more stringent stage-associated gene set was then defined to identify genes showing consistent transcriptional differences across both adjacent disease-stage comparisons. Increasing genes were required to show a significant ordinal-stage association, monotonic increase in group means, positive log-fold changes in both Early DKD versus Control and Advanced DKD versus Early DKD, and FDR < 0.05 in both contrasts. Decreasing genes were required to meet the corresponding criteria in the opposite direction. This procedure yielded the stringent stage-associated signature used for subsequent external validation.

#### 4.3.5. Leave-One-Early-DKD-Out Robustness Analysis

To assess whether the GSE142025 stage-associated findings were disproportionately influenced by any individual Early DKD sample, six deterministic leave-one-out analyses were performed, each excluding one of the six Early DKD samples while retaining all Control and Advanced DKD samples. Each iteration therefore comprised 35 samples, including nine Controls, five Early DKD samples, and 21 Advanced DKD samples. The ordinal-stage model and the three categorical pairwise limma contrasts were refitted independently in each iteration using the same modeling, empirical Bayes moderation, and multiple-testing procedures applied in the primary analysis.

Robustness was evaluated by comparing each leave-one-out result with the primary analysis in terms of the number and overlap of significant stage-associated genes, Jaccard similarity, correlations of gene-level ordinal-stage coefficients and moderated t statistics, and concordance of effect direction. Stability of monotonic classification and of the stringent 66-gene stage-associated signature was also assessed. In addition, the disease-side evidence supporting the 21 prioritized compound–DKD relationships was re-evaluated in each iteration, including ordinal-stage significance, Advanced-DKD-versus-Control significance and direction, and monotonic classification. These analyses were used to assess discovery-cohort stability and did not redefine the primary stage-associated gene sets or compound–DKD prioritization.

#### 4.3.6. Inferred-Sex Sensitivity Analysis

Individual-level clinical sex annotation was not available in the GEO metadata in a form that could be reliably mapped to the 36 deposited transcriptomic samples. An expression-based sex-inference analysis was therefore used as a sample-level sensitivity assessment rather than as a substitute for clinical sex information. Twelve sex-chromosome-associated genes, *XIST*, *KDM5D*, *RPS4Y1*, *USP9Y*, *DDX3Y*, *UTY*, *ZFY*, *EIF1AY*, *TMSB4Y*, *PRKY*, *NLGN4Y*, and *TXLNGY*, were used. Their expression values were standardized across samples, and two-cluster k-means clustering was performed with 100 random starts using a fixed random seed of 1. The cluster with the higher mean *XIST* expression was designated as XX-like and the complementary cluster as XY-like.

As a plausibility check, the inferred group-level distributions were compared with the male/female composition reported by Fan et al. [5]. The published distributions were 7/2 for Control, 5/1 for Early DKD, and 13/9 for the original 22-member Advanced DKD cohort. Because only 21 Advanced DKD samples were represented in the deposited GSE142025 series used here, exact subject-level correspondence with the original Advanced cohort could not be established.

For adjusted pairwise analyses, inferred-sex status was included as an additional covariate in a no-intercept limma design matrix containing disease-group and inferred-sex terms, with XY-like used as the reference level. The same three pairwise contrasts used in the primary analysis were then re-estimated with empirical Bayes moderation and Benjamini–Hochberg adjustment. For the ordinal sensitivity analysis, an additive model containing an intercept, ordinal DKD stage, and inferred-sex status was fitted across all genes using the same empirical Bayes and multiple-testing procedures. Adjusted monotonic classification was derived from the corresponding disease-group-plus-inferred-sex model. Compound–target-enrichment analyses were additionally repeated using the inferred-sex-adjusted stage-associated and monotonic gene sets. These analyses were used to assess sensitivity to expression-inferred sex composition; the primary disease models remained those specified above.

### 4.4. External Validation in Independent Human DKD Cohorts

#### 4.4.1. Validation Datasets and Preprocessing

Two independent human DKD transcriptomic datasets, GSE30528 [15] and GSE96804 [16], were obtained from GEO and used for external validation. GSE30528 comprised glomerular samples from nine individuals with DKD and 13 controls. The processed expression matrix contained 22,277 probe-level features across 22 samples. Probe annotations lacking a gene symbol or containing ambiguous multiple-gene assignments were excluded. The remaining 19,820 annotated probes represented 12,548 unique gene symbols; where multiple probes mapped to the same gene, expression values were collapsed to the gene level using limma::avereps.

GSE96804 comprised glomerular samples from 41 individuals with DKD and 20 controls. The processed GEO expression matrix initially contained 70,523 features across 61 samples. Features annotated with the category main were retained. The accompanying gene_assignment field was parsed to resolve gene symbols and Entrez identifiers. Individual assignments were considered valid when at least five annotation fields were present, the gene-symbol field was neither ---, NULL, nor empty, and the Entrez field contained a numeric identifier. A feature was retained only when the valid assignments resolved to a single unique gene symbol and a single unique Entrez identifier. This procedure yielded 23,458 mapped features corresponding to 22,140 unique gene symbols; multiple features assigned to the same gene were collapsed using limma::avereps.

#### 4.4.2. Differential-Expression Analysis in Validation Cohorts

Differential expression was analyzed independently in each validation cohort using limma. For each dataset, samples were represented as a two-level factor corresponding to Control and DKD, and a no-intercept design matrix was fitted to the gene-level expression data. A DKD-versus-Control contrast was evaluated using empirical Bayes moderation. *p* values were adjusted using the Benjamini–Hochberg procedure, with FDR < 0.05 considered statistically significant. No additional absolute log-fold-change threshold was imposed.

#### 4.4.3. Cross-Cohort Concordance and Replication of the Stringent Stage-Associated Signature

For genome-wide cross-cohort comparison, gene symbols shared between each validation dataset and the GSE142025 Advanced DKD-versus-Control analysis were identified. Effect-size concordance was assessed using the Pearson correlation between corresponding log-fold-change estimates. Directional concordance was additionally evaluated among genes reaching FDR < 0.05 in both the discovery and validation datasets. The shared comparison comprised 10,503 genes for GSE30528 and 14,883 genes for GSE96804.

The 66-gene stringent stage-associated signature defined in GSE142025 was then evaluated separately in each validation cohort. A stringent-signature gene was considered externally replicated when it was measurable in the validation dataset, reached FDR < 0.05 in the validation DKD-versus-Control comparison, and showed the same direction of expression change as the corresponding GSE142025 Advanced DKD-versus-Control effect. External replication was interpreted as support for the gene–DKD association and not as validation of any compound–target relationship.

The same replication criterion was additionally applied to each of the 21 prioritized compound–DKD relationships to assess the reproducibility of the corresponding gene–DKD association across the two external cohorts. Replication status was evaluated separately in GSE30528 and GSE96804 and then summarized according to whether a relationship replicated in either cohort or in both cohorts. Genes not represented in an external dataset were classified as unavailable rather than as nonreplicated, thereby distinguishing lack of measurement from failure to meet the replication criterion. As a conservative relationship-level sensitivity analysis, the previously prioritized relationships were also assessed under an additional requirement of replication in at least one independent cohort. This analysis evaluated robustness of the disease-evidence component only and did not alter the underlying compound–target evidence or generate a revised prioritized gene set. Because external replication was already incorporated into the original definitions of Tiers 3 and 4, this additional criterion was not statistically independent of the original prioritization for relationships in those tiers.

### 4.5. Integration and Prioritization of Compound–DKD Relationships

#### 4.5.1. Integration of Compound–Target and Human DKD Evidence

The predefined compound–target sets were integrated separately with the GSE142025 transcriptomic results by gene symbol. Only targets represented in the GSE142025 expression matrix were eligible for expression-based evaluation; targets not measurable in this dataset remained part of the predefined compound–target universes but were not assigned disease-expression statistics. For each measurable compound–gene relationship, the integrated evidence record included differential-expression estimates for Early DKD versus Control, Advanced DKD versus Control, and Advanced DKD versus Early DKD; the ordinal-stage-association statistic and FDR; monotonic expression status across the three disease groups; membership in the stringent stage-associated signature; and external-replication status in GSE30528 and GSE96804. Because targets shared by anserine and RRR-α-tocopherol represented distinct compound–gene relationships, they were retained separately for each compound during integration.

Compound–target evidence and DKD evidence were retained as distinct evidence dimensions throughout the integration. Thus, strong disease-associated transcription or external DKD replication did not alter the underlying classification of the compound–target evidence, and conversely, strong compound–target evidence did not itself constitute evidence of involvement in human DKD.

#### 4.5.2. Enrichment of Compound Targets in the Stage-Associated DKD Landscape

To determine whether the predefined target sets were preferentially represented among genes associated with DKD stage, enrichment was evaluated separately for anserine and RRR-α-tocopherol using one-sided Fisher exact tests with the alternative hypothesis of over-representation. The background universe comprised all 17,184 genes measurable in GSE142025. Two disease-gene definitions were tested independently: genes with a significant ordinal-stage association at Benjamini–Hochberg FDR < 0.05 and the subset of significant stage-associated genes additionally showing monotonic expression across Control, Early DKD, and Advanced DKD. For each compound and disease-gene definition, target versus nontarget status was cross-tabulated against membership versus nonmembership in the corresponding disease-associated gene set, and odds ratios (ORs) and exact *p* values were calculated. No formal statistical comparison between the enrichment estimates of the two compounds was performed.

#### 4.5.3. Evidence-Based Prioritization of Compound–DKD Relationships

Following integration, candidate compound–DKD relationships were prioritized using an investigator-defined four-tier evidence framework. The framework was designed to preserve differences in the nature of the available compound–target and human DKD evidence rather than combine heterogeneous evidence types into a numerical composite score. The four tiers therefore represented distinct evidence configurations and were not interpreted as a simple quantitative ranking from strongest to weakest. The written tier criteria were applied uniformly to all eligible compound–gene relationships without post hoc manual exceptions. Importantly, the prioritized relationships were established before the subsequent pathway and single-cell analyses and were not modified on the basis of those downstream results.

Tier 1 (“Human-experimental DKD candidate”) included relationships classified as having either direct human biochemical compound–target evidence or human functional experimental evidence, with the relationship retained as a candidate or functional mediator, provided that the target was measurable in GSE142025 and showed both significant differential expression in Advanced DKD-versus-Control and a significant ordinal-stage association at FDR < 0.05. Tier 2 (“Supportive translational candidate”) applied to candidate relationships supported by direct experimental evidence from a non-human or otherwise translationally limited context, again requiring measurability in GSE142025, significant Advanced DKD versus Control differential expression, and a significant ordinal-stage association.

For relationships supported only by computational compound–target evidence, prediction strength was ranked separately within each compound. Prediction percentiles were calculated from the within-compound ranks of the computational-prediction probabilities, with average ranks used for ties; values ≥ 0.75 were considered to fall within the upper quartile of the corresponding compound’s computational predictions. Tier 3 (“Balanced computational priority”) required an upper-quartile computational prediction, measurability in GSE142025, FDR < 0.05 for Advanced DKD versus Control, FDR < 0.05 for the ordinal-stage association, and replication in at least one of the two independent DKD cohorts. Tier 4 (“Disease-led computational priority”) comprised computationally supported relationships that replicated in both independent DKD cohorts but did not satisfy the complete Tier 3 balanced-priority criteria.

External replication within this prioritization framework used the same disease-replication definition described above: significance at FDR < 0.05 in the external DKD-versus-Control comparison together with concordant direction relative to the GSE142025 Advanced DKD-versus-Control effect. Accordingly, external replication strengthened the gene–DKD evidence dimension but did not upgrade the underlying compound–target evidence category. Application of these criteria yielded 21 prioritized compound–DKD relationships representing 20 unique genes; these relationships constituted the final gene-level prioritization and were carried forward unchanged into the downstream pathway, renal localization, and robustness analyses.

### 4.6. Pathway Analyses

#### 4.6.1. Disease-Associated Pathway Analysis

Pathway-level changes associated with DKD stage were evaluated using pre-ranked gene-set enrichment analysis (GSEA) [55]. All 17,184 genes analyzed in GSE142025 were ranked in decreasing order according to the moderated *t* statistic for the ordinal-stage coefficient obtained from the Control = 0, Early DKD = 1, and Advanced DKD = 2 model. Thus, positive ranking statistics represented genes whose expression tended to increase with increasing DKD stage, whereas negative statistics represented genes whose expression tended to decrease with increasing stage. Pathway enrichment was therefore interpreted as pathway association with DKD stage rather than as an effect of either compound.

As an initial broad functional analysis, the 50 Hallmark gene sets were obtained for Homo sapiens from the Molecular Signatures Database (MSigDB), release 2026.1.Hs [56], through msigdbr [57]. Gene symbols within each gene set were deduplicated, and pre-ranked GSEA was performed using fgseaMultilevel [58], with a minimum pathway size of 15 genes, a maximum size of 500 genes, and a fixed random seed of 123 for reproducibility. Pathways with a Benjamini–Hochberg-adjusted *p* value (FDR) < 0.05 were considered significant. The sign of the normalized enrichment score (NES) was used to distinguish pathways positively or negatively associated with increasing DKD stage.

#### 4.6.2. Reactome Pathway Analysis and Redundancy Reduction

For more detailed pathway-level integration, human Reactome gene sets [59] were obtained from the MSigDB C2 canonical-pathway collection, Reactome subcollection (CP:REACTOME), release 2026.1.Hs, using msigdbr. Unique gene symbols were retained within each pathway. Reactome pathways were analyzed using the same pre-ranked ordinal-stage statistic and fgseaMultilevel procedure, with pathway sizes restricted to 15–500 genes.

Because the Reactome collection contains extensive hierarchical and gene-content redundancy, the resulting pathway set was reduced using fgsea::collapsePathways. A total of 1083 Reactome pathways met the specified size criteria and were subjected to pathway collapsing with a *p*-value threshold of 0.05 to identify nonredundant representative pathways. This procedure yielded 129 representative Reactome pathways that were retained as the common pathway universe for subsequent compound–target enrichment and integrated pathway analysis.

#### 4.6.3. Compound–Target Reactome Over-Representation Analysis

Compound-associated pathway enrichment was evaluated independently from the disease GSEA. For this analysis, the predefined target sets were restricted to targets measurable in GSE142025 after excluding relationships classified primarily as direct compound handling/binding or handling/transport. This produced pathway-analysis target sets of 73 genes for anserine and 83 genes for RRR-α-tocopherol, including 11 genes shared between the two sets. The background universe comprised all 17,184 genes represented in the GSE142025 stage-ranking analysis. This background therefore tested over-representation relative to the measurable kidney transcriptome. Because the heterogeneous target-evidence sources did not share a single reproducible gene-level candidate universe, no alternative target-eligible background was imposed.

Over-representation was tested separately for each compound across the 129 retained Reactome pathways. For each pathway, genes were first restricted to the 17,184-gene background, and a 2 × 2 contingency table was constructed from compound–target membership and pathway membership. A one-sided Fisher exact test with the alternative hypothesis of over-representation was performed, and the resulting *p* values were adjusted across the 129 pathways for each compound using the Benjamini–Hochberg procedure. Pathways with FDR < 0.05 were considered significantly enriched for the corresponding compound–target set.

#### 4.6.4. Target-Confidence Sensitivity Analysis of Reactome Enrichment

To assess the sensitivity of the Reactome over-representation results to compound–target confidence, the analysis was repeated using the conservative restricted target sets defined above. Only restricted targets represented in the 17,184-gene GSE142025 background were eligible for the pathway sensitivity analysis. Reactome membership was evaluated using the same fixed set of 129 representative pathways established in the primary analysis; pathway collapsing was not repeated for the restricted target sets.

For each compound, one-sided Fisher exact tests were repeated across the same 129 pathways using the 17,184 measurable genes as the background universe, with Benjamini–Hochberg adjustment performed separately for each compound. Pathways with FDR < 0.05 were considered significantly enriched under the restricted target definition. The sensitivity analysis was used to determine which primary compound-specific and shared pathway-enrichment signals remained supported under the more conservative target-confidence criterion; it did not replace the primary pathway analysis or alter the previously prioritized compound–DKD relationships.

#### 4.6.5. Identification of Shared and Compound-Specific Pathway Relationships

In the primary target-set over-representation analysis, pathways reaching FDR < 0.05 for both compounds were classified as shared pathway-level convergence signals. This classification was based solely on significant compound–target enrichment and did not require the presence of a previously prioritized compound–DKD relationship. Pathways significant for one compound but not significant for the other were classified as compound-specific at the pathway-enrichment level. Importantly, non-significance for the second compound was not interpreted as evidence of absence of biological involvement.

Four of the retained Reactome pathways were significantly enriched for both compound–target sets and were therefore carried forward as shared pathway-level signals. After removal of these shared pathways, 12 statistically significant compound-specific pathways remained for each compound. For focused pathway integration and visualization, compound-specific pathways were further retained when they contained at least one of the 21 compound–DKD relationships that had been prioritized before pathway analysis. This yielded six anserine-specific and six RRR-α-tocopherol-specific pathways for the focused analysis. This prioritization-based retention step was applied only after the statistical pathway analyses and did not alter either the pathway-enrichment statistics or the previously established 21 compound–DKD relationships.

The previously prioritized compound–DKD relationships were subsequently mapped onto both the shared and retained compound-specific Reactome pathways to identify which prioritized targets were represented within the corresponding pathway-level context. Disease-stage NES values and compound–target over-representation were maintained as distinct evidence dimensions throughout interpretation: the former describes pathway association with increasing DKD stage, whereas the latter indicates enrichment of predefined compound targets within the pathway. Neither was interpreted as evidence that either anserine or RRR-α-tocopherol activates, inhibits, restores, or reverses the corresponding pathway.

### 4.7. Renal Single-Cell Transcriptomic Localization

#### 4.7.1. Dataset Processing and Quality Control

The human kidney single-cell RNA-sequencing dataset GSE209781, associated with the study by Lv et al. [60], was obtained from GEO and used to characterize the renal cellular distribution of the prioritized compound–DKD genes. The analysis included six donors, comprising three non-DKD samples (NM01–NM03) and three DKD samples (DKD01–DKD03). Single-cell data were processed in Seurat [61]. Cells were retained when they contained between 300 and 7000 detected genes (nFeature_RNA) and had mitochondrial transcript proportions below 30%. No upper threshold for total RNA counts and no hemoglobin-expression filter were applied. After quality control, 48,314 cells were retained for analysis.

Following quality control, RNA expression values were normalized using Seurat’s LogNormalize method with a scale factor of 10,000. Variable features were identified using the variance-stabilizing transformation (vst) method, retaining 3000 features, which were subsequently scaled and used for principal component analysis with 50 principal components. Donor-level datasets were then integrated using Seurat’s reciprocal principal component analysis (RPCA) integration workflow. Neighbor-graph construction and unsupervised clustering were performed using dimensions 1–30 of the integrated RPCA reduction, with a clustering resolution of 0.6. Uniform manifold approximation and projection (UMAP) visualization was generated using the same 30 dimensions. This procedure yielded 29 clusters.

#### 4.7.2. Cell-Type Annotation

Cluster marker genes were identified using Seurat’s FindAllMarkers function, retaining positive markers with a minimum detection fraction of 0.25 and a log-fold-change threshold of 0.25. Clusters were manually assigned to renal and immune cell populations on the basis of their transcriptional characteristics and cluster-level expression patterns. Clusters representing the same biological population were subsequently combined for localization analyses. One cluster showing a mixed/doublet-like transcriptional profile was excluded from subsequent localization analyses. This exclusion left 48,148 cells for localization analysis across the 17 retained renal and immune cell populations. No transcriptionally distinct podocyte population could be identified with sufficient confidence and, accordingly, no podocyte annotation was assigned.

The final annotation comprised 17 cellular populations: B cells, collecting-duct intercalated cells, collecting-duct principal cells, cycling immune cells, endothelial cells, fibroblasts, inflammatory monocytes, injured/transitional tubular cells, macrophage/dendritic cells, mast cells, neutrophils, natural killer (NK)/cytotoxic lymphocytes, pericyte/vascular smooth muscle cells, proximal tubule cells, T cells, thick ascending limb/distal nephron cells, and vascular/perivascular cells.

#### 4.7.3. Localization of Prioritized Compound–DKD Genes

The 21 prioritized compound–DKD relationships corresponded to 20 unique genes because *ACE2* was represented for both compounds. Expression of these 20 genes was summarized across each of the 17 retained cell populations. For every gene-cell-type combination, two descriptive metrics were calculated: mean normalized expression within the cell population and the percentage of cells with detectable expression. This produced a 20-gene × 17-cell-type localization matrix containing 340 gene-cell-type combinations.

The single-cell analysis was used exclusively to characterize the relative renal cellular localization of the 20 unique prioritized genes and was not interpreted as evidence of cell-specific pharmacological action by anserine or RRR-α-tocopherol. For visualization, average expression was standardized separately for each gene across the 17 cell populations, whereas the percentage of expressing cells was retained on its original scale. Accordingly, the resulting expression scaling supports comparisons of relative cellular localization within each gene and should not be interpreted as an absolute expression comparison between different genes.

#### 4.7.4. Donor-Aware Renal-Localization Robustness Analysis

To assess whether pooled renal-localization patterns were disproportionately influenced by individual donors, the six GSE209781 donors were analyzed separately and by deterministic leave-one-donor-out reaggregation. For each of the 20 unique prioritized genes, mean normalized expression and the percentage of expressing cells were summarized by donor and cell type. The pooled localization profiles were then recalculated after exclusion of each donor in turn, generating six leave-one-donor-out profiles for each gene. DKD and non-DKD donor groups were also aggregated separately and compared descriptively.

Localization robustness was assessed jointly from donor-level cell-type coverage, donor-resolved expression patterns, stability of the pooled localization profile across the six leave-one-donor-out analyses, and consistency of the broader cellular context between DKD and non-DKD aggregates. Robustness classifications were assigned from these complementary evidence components without imposing a post hoc numerical cutoff. These analyses were restricted to assessment of renal-localization stability; individual cells were not treated as independent biological replicates, and no inferential cell-type differential-expression testing was performed.

### 4.8. Data Visualization and Figure Generation

Graphical outputs were generated from the analysis objects and corresponding result tables without altering the underlying analytical results or selection criteria. Visualizations were produced primarily in R using ggplot2 and supporting packages appropriate to individual figure types, including pheatmap for expression heatmaps and eulerr for proportional target-set overlap visualization. Additional R graphics and layout utilities were used for specialized panels and multi-panel assembly.

For visualization of the stringent stage-associated transcriptional signature, expression values were standardized gene-wise across samples and displayed as row-wise z-scores, with samples ordered by Control, Early DKD, and Advanced DKD and genes grouped according to increasing or decreasing stage-associated expression rather than by unsupervised clustering. For single-cell localization plots, dot size represented the percentage of cells expressing each gene, whereas color represented average expression standardized separately for each gene across the annotated cell populations. This scaling was used to visualize relative cellular localization within a gene and was not interpreted as an absolute comparison of expression levels between different genes.

Multi-panel figures were assembled from the individual analytical panels while preserving their underlying data and graphical content. Figures were exported in publication-quality raster and/or vector formats, including TIFF, PNG, and PDF, as appropriate. Figure construction and assembly did not involve additional statistical filtering or post hoc modification of the predefined target sets, prioritized compound–DKD relationships, pathway results, or sensitivity and robustness results.

### 4.9. Statistical Analysis and Software

All computational and statistical analyses were performed in R version 4.6.1 [62]. Major packages used across the workflow included limma version 3.68.5, GEOquery version 2.80.0 [63], fgsea version 1.38.0, msigdbr version 26.1.1, Seurat version 5.5.1, dplyr version 1.2.1 [64], ggplot2 version 4.0.3 [65], pheatmap version 1.0.13 [66], and eulerr version 8.3.0 [67].

For bulk transcriptomic analyses, linear modeling and empirical Bayes moderation were performed using limma. Differential-expression and ordinal-stage-association *p* values were adjusted using the Benjamini–Hochberg procedure, with FDR < 0.05 defining statistical significance; no additional absolute log-fold-change cutoff was imposed. The ordinal-stage model encoded Control, Early DKD, and Advanced DKD as 0, 1, and 2, respectively. Pearson correlation coefficients were used descriptively to quantify concordance of gene-level log-fold-change estimates between GSE142025 and each external validation cohort.

Compound–target enrichment within the DKD transcriptional landscape and Reactome compound–target over-representation analyses were evaluated using one-sided Fisher exact tests with the alternative hypothesis of enrichment. The 17,184 genes measurable in GSE142025 constituted the background universe for both compound–target enrichment within the DKD transcriptional landscape and Reactome compound–target over-representation analyses. Reactome over-representation *p* values were adjusted using the Benjamini–Hochberg procedure across the 129 retained pathways for each compound.

Pre-ranked Hallmark and Reactome GSEA was performed using fgsea, with genes ranked by the moderated ordinal-stage *t* statistic. Multiple testing within the pathway analyses was controlled using the Benjamini–Hochberg FDR, with FDR < 0.05 considered significant. Single-cell gene-localization was summarized using average expression and the percentage of expressing cells; no inferential cell-type differential-expression testing was performed as part of the localization analysis. Donor-aware localization robustness was evaluated descriptively using donor-resolved summaries, six leave-one-donor-out reaggregations, and separate DKD and non-DKD aggregates; no individual cells were treated as independent biological replicates.

### 4.10. Use of Artificial Intelligence (AI)-Assisted Tools

OpenAI ChatGPT (OpenAI; GPT-5-series models accessed during 2026) was used as an AI-assisted tool during study planning, code drafting and troubleshooting, interpretation and organization of analytical outputs, figure planning, and manuscript drafting and language refinement. All analytical codes were executed by the author, and all analytical decisions, numerical results, figures, and interpretations were independently reviewed and verified by the author. Literature citations and factual claims were checked against the original sources. AI-generated output was not treated as primary evidence, and the author retained full responsibility for the study design, analyses, interpretation, and final manuscript.

## Data Availability

The publicly available human transcriptomic datasets analyzed in this study are available from the National Center for Biotechnology Information (NCBI) Gene Expression Omnibus (GEO) under accession numbers GSE142025, GSE30528, GSE96804, and GSE209781. The curated compound–target evidence and derived analytical results supporting the findings of this study are provided within the article and its Supplementary Materials. No new primary human datasets were generated in this study. The custom R scripts and essential fixed analytical inputs required to reproduce the analyses are publicly available on Zenodo (Version 1.0.0; DOI: 10.5281/zenodo.23372836).

## Supplementary Materials

The following supporting information can be downloaded at: https://www.mdpi.com/article/10.3390/ijms27209279/s1.

## Abbreviations

AI	Artificial intelligence
CID	Compound identifier
CKD	Chronic kidney disease
DKD	Diabetic kidney disease
FDR	False discovery rate
GEO	Gene Expression Omnibus
GPCR	G protein-coupled receptor
GSEA	Gene-set enrichment analysis
GST	Glutathione S-transferase
Hsp70	Heat shock protein 70
MMP	Matrix metalloproteinase
MSigDB	Molecular Signatures Database
NCBI	National Center for Biotechnology Information
NES	Normalized enrichment score
NK	Natural killer
OR	Odds ratio
PC1	Principal component 1
PC2	Principal component 2
PCA	Principal component analysis
PI3K	Phosphoinositide 3-kinase
RPCA	Reciprocal principal component analysis
SARS-CoV	Severe acute respiratory syndrome coronavirus
SEA	Similarity ensemble approach
TNF-α	Tumor necrosis factor alpha
UMAP	Uniform manifold approximation and projection
VEGF	Vascular endothelial growth factor
