# Supplementary material for: Distinct Molecular Relationships and Partial Pathway Convergence of Anserine and RRR-α-Tocopherol in Human Diabetic Kidney Disease
Source: Int J Mol Sci. 2026 Oct 19;27(20):9279. doi: 10.3390/ijms27209279 (PMC13705285; doi:10.3390/ijms27209279)
Supplement: Supplementary file 1 [file ijms-27-09279-s001.zip › Supplementary Files/Supplementary_Figure_S1_External validation of the stringent 66-gene stage-associated DKD signature in independent human cohorts.pdf]

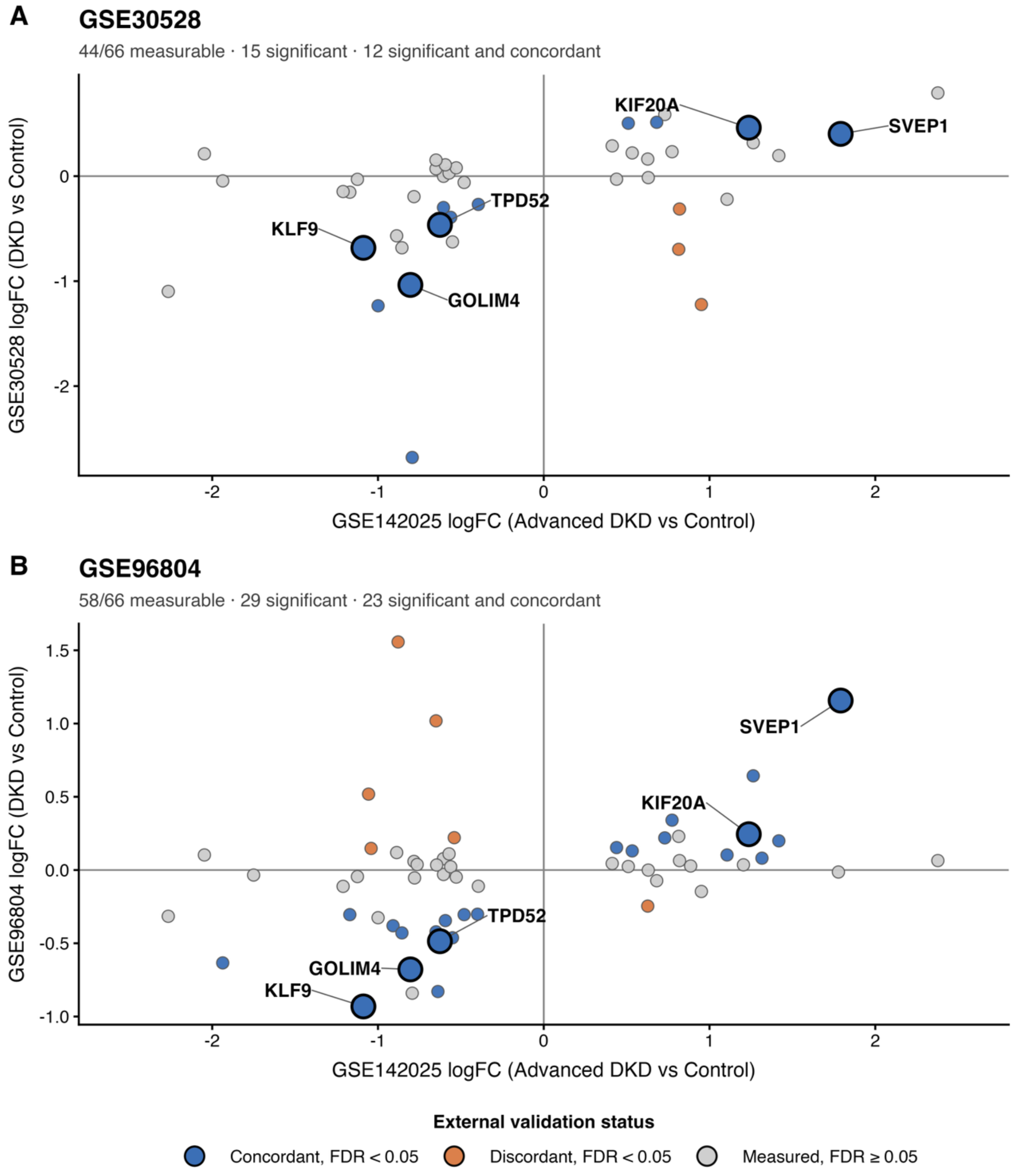

Supplementary Figure S1. External validation of the stringent 66-gene stage-associated DKD signature in independent human cohorts. Scatterplots compare gene-level differential-expression effects in the GSE142025 discovery cohort with those observed in the independent human DKD cohorts **(A)** GSE30528 and **(B)** GSE96804. The x-axis shows GSE142025 Advanced DKD versus Control log2 fold change, and the y-axis shows DKD versus Control log2 fold change in the corresponding external cohort. Only genes from the stringent 66-gene signature measurable in each external dataset are shown. In GSE30528, 44 of 66 genes were measurable, 15 had FDR < 0.05, and 12 met the predefined external-replication criterion; in GSE96804, 58 genes were measurable, 29 had FDR < 0.05, and 23 met this criterion. External replication was defined as FDR < 0.05 in the external DKD-versus-Control comparison together with concordant effect direction relative to GSE142025 Advanced DKD versus Control. Blue points indicate genes meeting this criterion, orange points indicate genes with FDR < 0.05 but discordant effect direction, and gray points indicate measurable genes with FDR ≥ 0.05. SVEP1, KIF20A, TPD52, KLF9, and GOLIM4, which met the replication criterion in both external cohorts, are highlighted and labeled. External replication supports reproducibility of the gene-DKD association and does not constitute validation of a compound-target relationship.
